# Examining the role of AI technology in online mental healthcare: opportunities, challenges, and implications, a mixed-methods review

**DOI:** 10.3389/fpsyt.2024.1356773

**Published:** 2024-05-07

**Authors:** Gilmar Gutierrez, Callum Stephenson, Jazmin Eadie, Kimia Asadpour, Nazanin Alavi

**Affiliations:** ^1^ Department of Psychiatry, Faculty of Health Sciences, Queen’s University, Kingston, ON, Canada; ^2^ Faculty of Education, Queen’s University, Kingston, ON, Canada; ^3^ Department of Psychology, Faculty of Arts and Sciences, Queen’s University, Kingston, ON, Canada; ^4^ Centre for Neuroscience Studies, Faculty of Health Sciences, Queen’s University, Kingston, ON, Canada; ^5^ OPTT Inc., Toronto, ON, Canada

**Keywords:** online psychotherapy, artificial intelligence, mental health, machine learning, artificial neural networks

## Abstract

**Introduction:**

Online mental healthcare has gained significant attention due to its effectiveness, accessibility, and scalability in the management of mental health symptoms. Despite these advantages over traditional in-person formats, including higher availability and accessibility, issues with low treatment adherence and high dropout rates persist. Artificial intelligence (AI) technologies could help address these issues, through powerful predictive models, language analysis, and intelligent dialogue with users, however the study of these applications remains underexplored. The following mixed methods review aimed to supplement this gap by synthesizing the available evidence on the applications of AI in online mental healthcare.

**Method:**

We searched the following databases: MEDLINE, CINAHL, PsycINFO, EMBASE, and Cochrane. This review included peer-reviewed randomized controlled trials, observational studies, non-randomized experimental studies, and case studies that were selected using the PRISMA guidelines. Data regarding pre and post-intervention outcomes and AI applications were extracted and analyzed. A mixed-methods approach encompassing meta-analysis and network meta-analysis was used to analyze pre and post-intervention outcomes, including main effects, depression, anxiety, and study dropouts. We applied the Cochrane risk of bias tool and the Grading of Recommendations Assessment, Development and Evaluation (GRADE) to assess the quality of the evidence.

**Results:**

Twenty-nine studies were included revealing a variety of AI applications including triage, psychotherapy delivery, treatment monitoring, therapy engagement support, identification of effective therapy features, and prediction of treatment response, dropout, and adherence. AI-delivered self-guided interventions demonstrated medium to large effects on managing mental health symptoms, with dropout rates comparable to non-AI interventions. The quality of the data was low to very low.

**Discussion:**

The review supported the use of AI in enhancing treatment response, adherence, and improvements in online mental healthcare. Nevertheless, given the low quality of the available evidence, this study highlighted the need for additional robust and high-powered studies in this emerging field.

**Systematic review registration:**

https://www.crd.york.ac.uk/prospero/display_record.php?RecordID=443575, identifier CRD42023443575.

## 1 Introduction

The management of mental illness represents an important healthcare challenge. In the United States and Canada, around one fifth of adults experience mental illness, yet less than 50% have access to or are receiving treatment (1–6). Some barriers that have been reported include negative attitudes towards seeking help for mental illness, lack of availability, and long wait times due to mental health professional shortages, elevated costs, geographical and mobility factors. (7–12). These barriers were exacerbated by the COVID-19 pandemic, further highlighting the need for accessible mental healthcare solutions and more efficient systems (7–12). Thus, online mental healthcare solutions, such as digital mental healthcare interventions (DMHIs), have emerged as promising alternatives leveraging the benefits of online platforms to overcome the barriers inherent in in-person services (8, 13–16). Another recent solution has involved the use of artificial intelligence (AI), as a powerful tool capable of automating several aspects of the healthcare process, from healthcare monitoring and triage to diagnosis, risk assessment, and even treatment delivery (6, 17–23). The research and validation of online mental health alternatives and AI applications have experienced significant growth and development in recent years (6, 8, 13–23). However, the study of the applications of AI in mental healthcare remains in its infancy (6, 20).

Online mental healthcare presents several advantages compared to its in-person counterpart, notably additional privacy, and the ability to access healthcare anywhere with an internet connection (11, 24). Additionally, studies have shown that despite some concerns over the strength of the therapeutic relationship, online mental healthcare has similar effectiveness as in-person options for the management of mental health conditions (11, 24). For instance, a study by Alavi et al. (2023) showed that an online cognitive behavioral therapy program (eCBT) for depression had similar effectiveness and attrition rates as its in-person counterpart, with a medium to large effect size in the management of depression symptoms (25). Nevertheless, despite the promising results obtained with online mental healthcare, some gaps and barriers remain, limiting its use (5, 15, 24, 26). For instance, similarly to in-person options, diagnosis-specific online mental healthcare often requires a diagnosis and triage process by a healthcare professional. This can result in similar long wait times as that experienced by individuals seeking in-person care (5, 15, 24, 26). Monitoring of treatment progress, risk assessment, personalization of the therapy experience, and training of new therapists also pose a challenge to online mental healthcare (5, 15, 19, 20, 24, 26). Especially in the case of fully self-guided online psychotherapy, the lack of monitoring, risk assessment and personalization may place the patient at an increased risk of dropping out from the treatment or experiencing an exacerbation of psychiatric symptoms (20, 24). AI technology can help address these issues by supporting a versatile, scalable, and powerful approach that can analyze and respond to large amounts of data and adapt to individual healthcare needs (6, 17–23).

In recent years AI has garnered significant attention for a variety of applications, showcasing its significant versatility and ability to integrate and enhance a range of services and fields of study (6, 17–23). In healthcare, AI is now being used to aid in clinical decision-making, facilitating disease detection and monitoring, optimizing medical management, and even the discovery of novel therapies (20, 22, 23). This is possible due to AI’s ability to process and learn from large amounts of data either through a supervised or unsupervised approach (6, 19). In the supervised approach, the AI algorithm uses known outputs (training sets) to develop patterns to predict outcomes in other datasets, while in the unsupervised approach, AI learns from unknown outputs to find patterns in the data (6, 19). The use of ML algorithms, which can analyze large datasets, has garnered significant attention in online mental healthcare with several models now being proposed in this field including: probabilistic latent variable model, linear ML model, random forest model, Latent Dirichlet Allocation (LDA) topic models, elastic net model, inductive logic programming, decision tree model, support vector machine, deep learning (DL), and artificial neural networks (ANN) (27–33). However, several concerns have been raised in terms of the validity, generalizability, and reliability of the results obtained using ML, which can be impacted by insufficient or not representative training datasets, improper model fitting or hyperparameter fine-tuning, improper handling of training data sets resulting in data leakage, lack of validation and reproducibility assessments, among others (34–37). Therefore, to support the robustness of studies using ML algorithms, recent guidelines have proposed six essential elements: justification of the need to use ML, adequacy of the data, description of the algorithm used, results including model accuracy and calibration, availability of the programming code, and discussion of the model’s internal and external validation (34–37).

If carefully implemented, ML can produce results with a precision that can rival or surpass seasoned clinicians (20). For instance, recent advances in AI-powered tools have shown similar or better sensitivity for detecting minuscule deviations from normal anatomy, compared to a human assessor (20, 38–40). In contrast, the application of AI in mental healthcare remains in its infancy (6, 20). Nevertheless, recent studies have shown promising results of AI applications including suicide risk assessment and risk prediction, identification of mental illness predictors, mental health monitoring, psychoeducation and psychotherapy delivery, therapist training, mental healthcare personalization, mental health triage and decision-making, and promoting treatment engagement (6, 17–23). In terms of therapy content, this includes CBT, acceptance and commitment therapy (ACT), dialectic behavioral therapy, mindfulness, and supportive therapy (6, 41). However, because of the novelty of this technology, there are several unanswered questions and considerations such as limitations in the interpretation of language, biases in the interaction with patients from diverse backgrounds, and unanswered ethical, patient safety, and health policy considerations (6, 17–23).

The benefits provided by AI technologies can revolutionize online mental healthcare in many ways, supporting shorter wait times, enhanced accessibility, personalization, and engagement. Therefore, to supplement the available literature, this review presents the most comprehensive and first mixed-methods review, which implements both meta-analytic and network meta-analytic approaches to synthesize the available evidence on the applications of AI in online mental healthcare. This exhaustive review considered a broad range of AI applications from triage processes, and the delivery of psychoeducation and psychotherapy, to the monitoring of therapy progress and the ability to support therapy engagement. Based on the available evidence, we hypothesized that the results of this mixed methods review will support the implementation and applications of AI technology in online mental healthcare. Hence, applying a mixed methods review approach, we aimed to determine the impact that AI technologies are having on online mental healthcare, and the challenges and important considerations of this technology in clinical practice. Finally, future directions and recommendations will be discussed to help guide the development and implementation of AI technologies in online mental healthcare.

## 2 Methods

### 2.1 Protocol and registration

We registered this mixed methods systematic review with PROSPERO (CRD42023443575, https://www.crd.york.ac.uk/prospero/display_record.php?RecordID=443575) and followed the Preferred Reporting Items for Systematic Reviews and Meta-analysis (PRISMA) guidelines, including the extension for NMAs (42–44).

### 2.2 Data availability statement

The raw data supporting the conclusions of this article will be made available by the authors, without undue reservation.

### 2.3 Search strategy

We developed a comprehensive search strategy following the approach taken by previous reviews on online mental healthcare and AI applications in the field (6, 17–23, 45–49). The review question and article eligibility and exclusion criteria was defined by using a populations-interventions-comparators-outcomes-study (PICOS) design framework (50). The Medical Subject Headings (MeSH) terminology and specific keywords included terms related to AI, applications of AI, and online mental healthcare including different modalities of psychoeducation and psychotherapy can be found in **Supplementary Material 1**. Then, we searched MEDLINE, CINAHL, PsycINFO, EMBASE, and Cochrane for relevant articles. The search was limited to articles written from inception of the searched terms until October 2023. We searched the bibliographies of previous reviews in the field and included articles to identify additional studies that may not have been identified by our systematic search strategy.

### 2.4 Study selection and eligibility

We conducted the review on COVIDENCE, a web-based systematic review manager (51). Four co-authors (GG, CS, JE, and KA) independently screened the identified articles considering the eligibility and exclusion criteria. Two votes were required to approve any screening decisions, and conflicts or disagreements were resolved by consensus between the co-authors involved in the decision or a third co-author. The eligibility criteria included case studies, observational studies, open-label trials, and randomized controlled trials (RCTs) written in English on the applications of AI-augmented intervention and AI tools and algorithms in online mental healthcare. AI-augmented interventions correspond to any online mental healthcare intervention that integrates AI technology in some capacity (i.e., delivery, monitoring, assessment, etc.) and AI tools and algorithms correspond to any AI based analysis of online mental healthcare data. Exclusion criteria included review studies, editorial comments, grey literature, secondary analysis of data, and protocols.

### 2.5 Data extraction process

Four reviewers (GG, CS, JE, and KA) independently extracted the selected articles on COVIDENCE. Two votes were required to approve any extraction decisions, and conflicts or disagreements were resolved by consensus between the co-authors involved in the decision or a third co-author. We extracted: name of the study, authors, sample size, demographic information, study location, intervention characteristics (modality, frequency, focus, application of AI, comparators), outcome measures, treatment duration and follow-up. For outcome measures, we followed two extraction approaches based on the type of the articles. For RCTs, observational cohort, and non-randomized experimental studies, we extracted pre- and post-intervention outcomes for all the study arms. In preparation for this step, through consensus amongst the co-authors we identified depression symptom reduction and anxiety symptom reduction as the most common outcome measures in the included studies. We focused on the extraction of these outcome measures to avoid underpowered meta-analytic comparisons. For studies reporting on the results of large dataset analysis using ML algorithms, data extraction followed the recent research guidelines and standards for ML studies, which recommend that these studies report on the adequacy of the data for the intended outcomes, model training and fine-tuning, features analyzed, validation, interpretability, and code and data availability (34–37). Additionally, we extracted the clinical and practical insights and implications that the studies obtained and discussed regarding the implementation of ML. These studies were not included in the meta-analysis or network meta-analysis calculations, and their results were only included and discussed in a narrative fashion.

### 2.6 Risk of bias within studies

Four reviewers (GG, CS, JE, and KA) independently appraised the quality of the selected articles using the Cochrane Risk of Bias Tool (RoB2) (52). Two votes were required to approve any appraisal decisions, and conflicts or disagreements were resolved by consensus between the co-authors involved in the decision or a third co-author. The RoB2 considers high, unclear, or low risk of bias for six domains: randomization, allocation concealment, blinding of participants and evaluators, incomplete outcome reporting, and selective reporting (52). The randomization, allocation concealment and blinding of participants and evaluators domains are more closely related to the assessment of RCTs. Thus, to account for the inclusion of other study types we also included selection, confounding, information, allegiance, and adherence bias assessments. Selection bias occurs when the researcher can influence who gets recruited to the study. Confounding bias occurs when extraneous or unaccounted elements could have impacted the outcomes assessed by the study. Information bias occurs when the outcomes are not adequately measured with validated tools. Allegiance bias corresponds to the relation between the developer of the studied treatment and the researchers, and adherence bias refers to any changes or deviations in the protocol. The risk of bias in each study was considered to be high if any of the assessed domains scored high for risk of bias, or if they scored unclear on two or more domains (52, 53).

### 2.7 Risk of bias across studies

We assessed the risk of bias across studies or certainty of the evidence using the Grading of Recommendations Assessment, Development and Evaluation (GRADE) guidelines (54). The quality of the evidence was downgraded if the risk of bias within studies, publication bias, imprecision in outcomes, indirectness or heterogeneity were considered to be high (54). We analyzed publication bias using funnel plots (55) and Rosenthal’s fail-safe N. These techniques show the number of hypothetical null results that would be required to make the results of the analysis insignificant (56). Imprecision referred to the significance of the reported results using a 95% confidence interval (95%CI) and the appropriateness of the sample size to achieve a power of 0.8, and 95%CI (54, 57, 58). Indirectness is assessed by considering the applicability of the outcomes, the use of surrogate outcomes, and the number of indirect comparisons (54). We calculated transitivity or heterogeneity using τ^2^ (the total variation) and *I^2^* (the percentage of τ^2^ not related to random error) with higher values being associated with higher heterogeneity between studies (43, 59–62).

### 2.8 Summary measures

We used Cohen’s *d* standardized mean differences (SMD or d) to summarize continuous outcomes (e.g. symptom severity), and risk ratios (RRs) and odds ratios (ORs) for dichotomous data (e.g. dropouts and treatment response) (53, 63, 64). The outcome measures were reported using a 95% CI to determine statistical significance (65).

### 2.9 Planned methods of analysis

This mixed-methods review presented two types of analysis to maximize the comprehensive assessment of the identified articles: meta-analysis and network meta-analysis (66). This approach accounted for the scarcity of articles in this field and supported the generation of insights about the current state of AI technology and its applications to online mental healthcare. We analyzed four treatment outcomes: “Main effects” considered all the primary outcomes reported by the relevant included studies regardless of type of symptom management. We focused on main effects to support a more robust assessment of the impact of AI-augmented interventions on non-specific mental health symptoms, and to avoid underpowered estimates due to the scarcity of available studies. “Depression” considered only interventions specific to the management of depression symptoms and changes in depression symptoms severity scores. “Anxiety” considered only interventions specific to the management of anxiety symptoms and changes in anxiety symptoms severity scores. And “Treatment dropouts” considered all reported participant dropouts in the included studies. Following this outcome analysis approach, all the studies presenting pre- and post-intervention data resulting from the implementation of AI-augmented interventions, were analyzed using a meta-analytic approach. This analysis supported the understanding of the effect of AI-augmented interventions in the management of psychiatric symptoms. Then, only the RCTs presenting post-intervention psychiatric symptoms data using validated scales were analyzed using a network meta-analytic (NMA) approach. This analysis supported a deeper exploration of the effects of the available AI-augmented interventions in relation to other interventions in the field through direct and indirect comparisons (67). Articles reporting on the results of large data set analysis using ML algorithms were not included in the meta-analysis or network meta-analysis calculations.

Missing data was collected by contacting the authors when appropriate, otherwise, this data was excluded from the analysis. For both meta-analysis and NMA, we employed a random-effects model to calculate the SMD or RR for relevant outcomes in the included studies, and heterogeneity using τ^2^ and I^2^ with the *meta* package in R version 3.5.3, computed using RStudio and the NMA tool, Meta Insight (59, 61, 68–70). We employed a random-effects model to account for study heterogeneity (I^2^>50%, and to accommodate different types of measurement for the same outcome (e.g., different assessment scales for depression) (59, 61). We also plotted forest plots for each outcome measure. For the NMA, we employed the assumption that the participants would have a similar probability of being allocated to any available treatment, meaning that the network was jointly randomizable (59, 71). The NMA league plots were used to present a more comprehensive assessment of all available direct and indirect head-to-head comparisons and to rank the interventions based on the desirability of their effect compared to other interventions. Additionally, we analyzed inconsistency to determine the goodness of fit of the NMA model (p>0.05 indicates network consistency) (68, 71, 72). Finally, treatment dropouts and any identified side effects were presented as a proportion for all studies. For RCTs reporting treatment dropouts, we employed an NMA approach as detailed above to obtain a deeper understanding of the attrition rates among the identified AI-augmented interventions.

## 3 Results

### 3.1 Study selection

**Figure 1:**
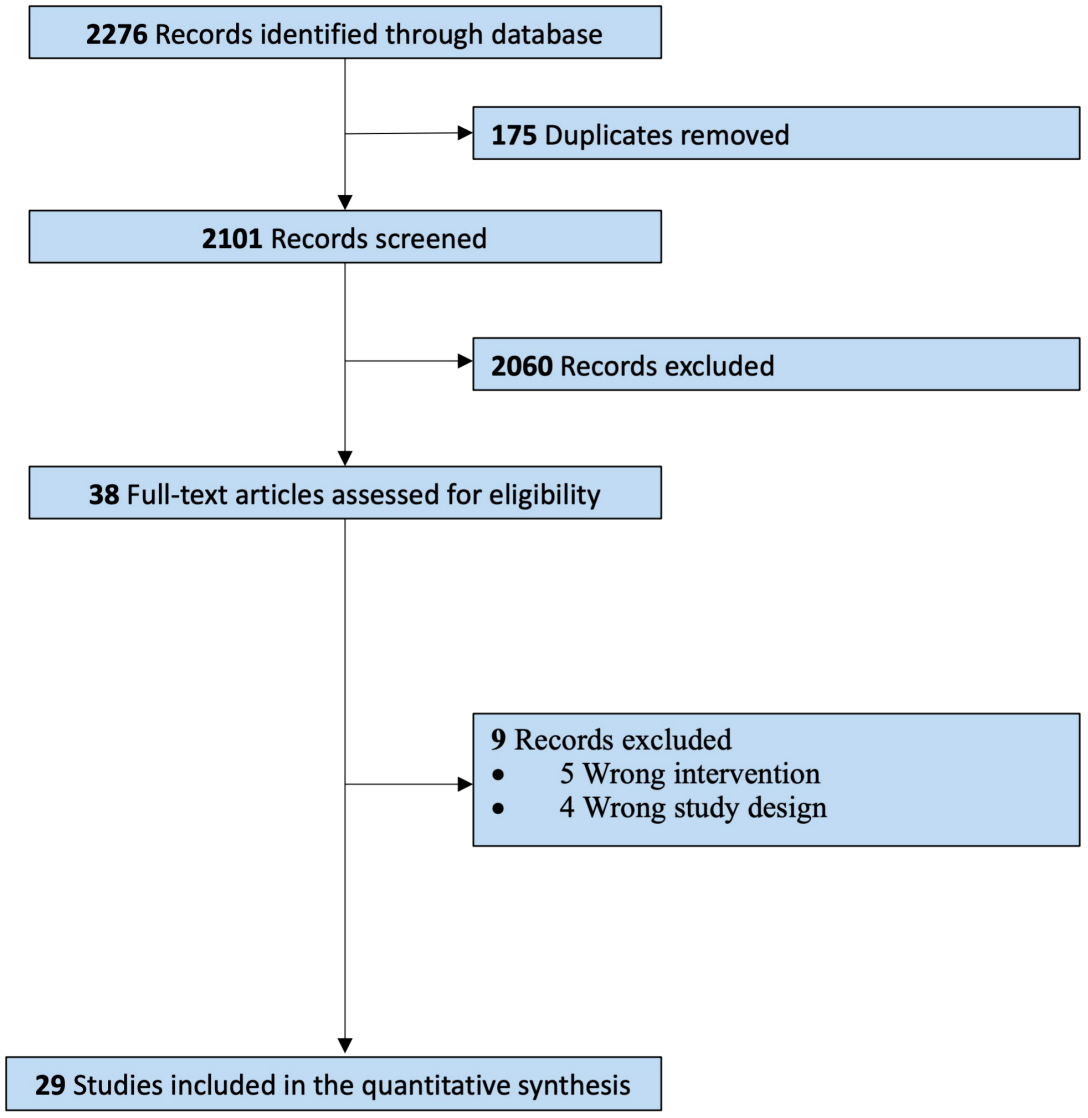
PRISMA guidelines – Study selection process.

Our systematic search identified a total of 2276 citations from inception to October 2023 (**Figure 1**). From these, 38 reports were assessed for full-text assessment, and 29 studies met the eligibility criteria for inclusion in this review. 9 records were excluded due to wrong intervention, and wrong study design. Kappa Interobserver agreement was determined to be good (k = 0.78), and disagreements were solved by consensus.

### 3.2 Study characteristics

**Figure 2:**
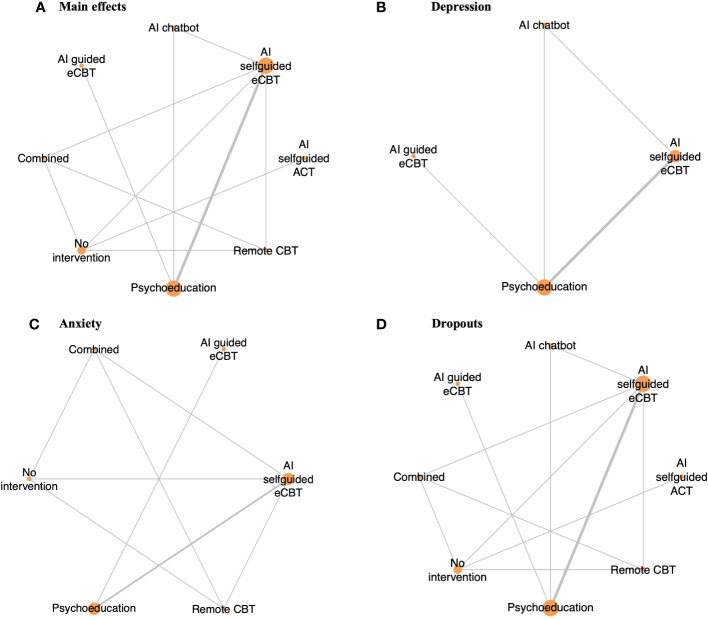
Network plot of RCTs included in this review, comparing the identified interventions (“AI self-guided eCBT”: AI agent delivering eCBT with no therapist guidance; “AI guided eCBT”: AI agent delivering eCBT with asynchronous therapist guidance; “Remote CBT”: Standard CBT delivered over the phone; “AI self-guided ACT”: AI agent delivered ACT with no therapist guidance; “Combined”: Remote CBT and AI self-guided eCBT; “AI chatbot”: AI agent providing general conversational responses not associated with CBT or therapy; “Psychoeducation”; and “No intervention”). Circle sizes represent the aggregated sample size relative to each intervention, and the thickness of the lines represents the number of studies comparing the respective interventions. **(A)** Main effects of all the included RCTs (n=6), **(B)** Depression scores in RCTs specific to the study of interventions for depression symptoms (n=4), **(C)** Anxiety scores in RCTs specific to the study of interventions for anxiety symptoms (n=4), **(D)** RCTs reporting participant dropouts (n=6).

The identified studies (n=29) were mostly conducted in the USA (n=10), Sweden (n=6), and the UK (n=6). The studies included a diverse sample of adults (n=22), college students and young adults (n=4), anonymous users (n=2), and adolescents (n=1) (28–33, 41, 73–94). Eight studies reported on the ethnicity of the participants (30, 32, 41, 74, 76, 77, 80, 83), and in most of the studies between 31% and 91% of the participants identified as White. The studies focused on a population with moderate psychiatric symptoms including depression, anxiety, obsessive-compulsive disorder (OCD), insomnia, stress, body dysmorphic disorder (BDD), opioid misuse, social anxiety disorder (SAD), and bipolar personality disorder. Most studies (n=20) included >50% of female participants, with n=4 studies not reporting the percentage of males or females in their study (31, 33, 84, 90). The selected studies reported on several AI-augmented interventions modalities and AI tools and algorithms including: AI-self-guided eCBT: AI agent or chatbot delivering eCBT with no therapist guidance (n=7), AI-guided eCBT: AI agent or chatbot delivering eCBT with asynchronous therapist guidance (n=1), AI-modified eCBT: AI based modifications to eCBT program (n=3), AI-self-guided ACT: AI agent or chatbot delivered acceptance and commitment therapy (ACT) with no therapist guidance (n=1), Combined: Remote CBT and AI-self-guided eCBT combined (n=1); AI prediction of treatment dropout (n=2), AI identification of useful therapy aspects (n=1), AI identification of themes in patients’ utterances and written assignments (n=2), AI matching patients to appropriate treatments (n=1), AI prediction of short and long-term response (n=9), and AI prediction of treatment engagement and adherence (n=2). The included RCTs, observational cohort and non-randomized experimental studies included the following comparators: no intervention (n=2), remote CBT: standard CBT program delivered over the phone (n=1), psychoeducation (n=4), AI chatbot: regular interactions with an AI chatbot not trained or designed to deliver therapeutic content (n=1) (**Table 1**). These studies assessed the following outcomes: main effects (n=10), depression symptoms (n=7), anxiety symptoms (n=4) and treatment dropouts (n=13) (**Table 2**). On average the interventions were 7.33 (SD=4.16) weeks long, and 10 studies reported follow-up measurements. The length of the follow-up was on average 30.44 (SD=29.21) weeks long. **Figure 2** presents all the head-to-head comparisons between the AI-augmented interventions and comparators as a network developed using NMA for the four main studied outcomes (main effects, depression, anxiety, and treatment dropouts).

**Table 1:** Characteristics of the included studies.

Study name	Country	Sample	Outcome measures	Study arms	N total per arm	Mean age (SD)	%Female	Ethnicity	Attrition (%) per arm	Number of sessions	Duration of intervention	Post intervention follow up
Randomized Controlled trials												
Anthony et. al. 2020	USA	76 adult individuals recruited from level 1 trauma centre for traumatic upper or lower fracture for operative fixation	Opioid table use Post-operative PROMIS pain intensity score Post-operative PROMIS pain interference score Post-operative PROMIS Emotional distress/Anxiety score	1. AI self-guided ACT for opioid addiction 2. No intervention	1. 38 2. 38	1. 45.5 (15.9) 2. 48.7 (14.6)	1. 52% 2. 48%	1. White: 88% Black: 10% Asian: 2% Latino: 0% 2. White: 88% Black: 10% Asian: 0% Latino: 2%	1. 9.5% 2. 5.0%	1. Twice per day mobile messages for 2 weeks 2. NA	2 weeks	NA
Danieli et. al. 2022	Italy	45 adult individuals with stress symptoms and mild-to-moderate anxiety	SCL-90-R PSS OSI PHQ-8 GAD-7 Satisfaction/5 Usefulness/5	1. Remote CBT for mild to moderate anxiety 2. Remote CBT + AI self-guided eCBT (TEO) or mild to moderate anxiety 3. AI self-guided eCBT (TEO) for mild to moderate anxiety 4. No intervention	1. 15 2. 12 3. 8 4. 10	1. 54.08 (4.11) 2. 55.17 (3.69) 3. 55.63 (4.50) 4. 57.20 (7.96)	1. 73% 2. 83% 3. 75% 4. 80%	Not reported	1. 6.3% 2. 25.0% 3. 20.0% 4. 33.3%	8 weekly lessons	8 weeks	3 months
Fitzpatrick et. al. 2017	USA	70 college students experiencing symptoms of depression and anxiety	PHQ9 GAD7 PANAS Acceptability/5 Usability/5	1. AI self-guided eCBT (Woebot) for depression and anxiety 2. Psychoeducation	1. 31 2. 25	1. 22.58 (2.38) 2. 21.83 (2.24)	1. 79% 2. 55%	1. White: 82% Latino: 6% Other: 12% 2. White: 75% Latino: 8% Other: 17%	1. 8.8% 2. 30.6%	1. Up to 20 sessions 2. NA	2 weeks	NA
Forsell et. al. 2019	Sweden	251 adult individuals experiencing symptoms of insomnia	ISI Score Client satisfaction questionnaire	1. Guided eCBT for insomnia + regular therapist check-ins (At risk of treatment failure) 2. Guided eCBT for insomnia (Not at risk or treatment failure) 3. Guided eCBT for insomnia (At risk of treatment failure)	1. 51 2. 50 3. 149	1. 46.2 (12.5) 2. 47.8 (13.9) 3. 43.4 (14.3)	1. 76.5% 2. 65.8% 3. 70.6%	Not reported	1. 3.9% 2. 0.0% 3. 2.0%	9 weekly sessions (randomization of at-risk patients on week 4 to continue their current eCBT program or receive regular therapist check-ins)	9 weeks	NA
Fulmer et. al. 2018	USA	75 college students experiencing symptoms of depression and anxiety	PHQ9 GAD7 PANAS User satisfaction and engagement	1. AI guided eCBT (Tess) for depression and anxiety with daily check-ins 2. AI guided eCBT (Tess) for depression and anxiety with biweekly check-ins 3. Psychoeducation	1. 26 2. 24 3. 24	1. 24.1 (5.4) 2. 22.2 (2.8) 3. 22.5 (4.0)	1. 71% 2. 73% 3. 67%	1. White: 54% Asian: 46% Black: 0% Other: 0% 2. White: 31% Asian: 69% Black: 0% Other: 0% 3. White: 46% Asian: 33% Black: 8% Other: 13%	1. 0.0% 2. 0.0% 3. 4.0%	1. Unlimited access for 2 weeks 2. Unlimited access for 4 weeks 3. Unlimited access for 4 weeks	1. 2 weeks 2. 4 weeks 3. 4 weeks	NA
He et. al. 2022	China	148 young adults with depression symptoms during other COVID19 pandemic	PHQ-9 WAQ Acceptability Scale (AS)	1. AI self-guided eCBT (XiaoE) for depression 2. Psychoeducation 3. AI Chatbot once a day (Xiaoai)	1. 49 2. 49 3. 50	1. 18.80 (0.89) 2. 18.92 (0.84) 3. 18.64 (0.90)	1. 36.7% 2. 36.7% 3. 38.0%	1. Han Chinese: 89.8% non-Han Chinese: 10.2% 2. Han Chinese: 89.8% non-Han Chinese: 10.2% 3. Han Chinese: 98.0% non-Han Chinese: 2.0%	1. 8.2% 2. 20.4% 3. 18.0%	1. 7 modules 2. Unlimited access for 1 week 3. Interactions once a day	1 week	1 month
Klos et. al. 2021	Argentina	181 college students experiencing symptoms of anxiety and depression	PHQ-9 GAD-7	1. AI self-guided eCBT (Tess) for depression and anxiety 2. Psychoeducation	1. 99 2. 82	25.5 (10.61)	87.2%	Not reported	1. 60.6% 2. 58.5%	Unlimited access for 8 weeks	8 weeks	NA
Morrison et. al. 2017	UK	77 adult users of Healthy mind to manage symptoms of stress	Usage metrics	1. Self-guided eCBT (Healthy mind) for stress with AI notifications 2. Self-guided eCBT (Healthy mind) for stress with daily notifications 3. Self-guided eCBT (Healthy mind) for stress with occasional notifications	1. 25 2. 19 3. 33	35.94 (10.54)	62%	Not reported	24.7%	Unlimited access during intervention period	2 weeks	NA
Non-randomized experimental studies												
Burns et. al. 2011	USA	8 adults individuals with major depressive disorder	Contextual data Website usage Mobile application training Accuracy predictions MINI PHQ9 QIDS-C GAD-7	Guided behavioral therapy + AI momentary sensing (Mobilyze) for depression	8	37.4 (12.2)	87.5%%	White: 88% Latino: 13%	12.5%	9 didactic lessons	8 weeks	NA
Frias et. al. 2021	Spain	25 adult individuals diagnosed with borderline personality disorder	BSL-23 DERS BDI System Usability Scale	AI self-guided eCBT (B-RIGHT) for borderline personality disorder	25	35.80 (9.90)	84%	Not reported	0.0%	Unlimited access for intervention period	1 month	NA
Observational cohort studies												
Binik et. al. 1988	USA	10 adult couples (20 participants total)	Reactions and perceptions of Sexpert	AI therapy (Sexpert) for sexual dysfunction	20	Not reported	50%	Not reported	0.0%	60 - 90 minutes session	1 session	NA
Bremer et. al. 2020	Germany and USA	151 adult individuals who participated in eCBT for insomnia (Sleep Healthy Using the Internet)	Prediction of user dropout from an online behavioral counselling program using a machine learning approach	Self-guided eCBT (SHUTi) for insomnia	151	43.3 (11.6	71.90%	White: 83.8% Black: 6.9% Asian: 4.0% Other: 5.3%	39.7%	7 weekly lessons	7 weeks	12 months
Inkster et. al. 2018	UK	129 anonymous global users reporting symptoms of depression	PHQ9 Engagement effectiveness Engagement efficiency	1. AI self-guided eCBT for depression - High users (used Wysa 3 or more days) 2. AI self-guided eCBT for depression - Low users (used Wysa 2 days)	129	Not reported	Not reported	Not reported	N/A	Multiple interactions with Wysa over 2 weeks	2 weeks	NA
Meheli et. al. 2022	USA	2,194 anonymous users reporting chronic pain and associated health conditions in their conversations with the mental health app	Textual snippets from users Tool usage data App usage data PHQ-9 GAD-7	AI self-guided eCBT (Wysa) for depression	2,194	Not reported	Not reported	Not reported	NA	Multiple interactions with Wysa during the study period (October 2020 to October 2021)	Multiple interactions with Wysa during the study period (October 2020 to October 2021)	NA
Machine learning studies												
Study name	Country	Sample	Outcome measures	Study arms	Duration of intervention	Post intervention follow up	Machine learning model	Data adequately explained?	odel training and fine-tuning	Features analyzed	Validation and interpretability	Code and data availability
Chien et. al. 2020	UK	54,604 adult individuals (age, %female, and ethnicity not reported) who participated in the Space From Depression and Anxiety treatment program from January 31, 2015, to March 31, 2019,	Identification of engagement subtypes using a machine learning approach	Guided eCBT for depression and anxiety (8 core modules)	14 weeks	NA	Probabilistic latent variable models: Hidden Markov model and Latent variable mixed model	Yes	Model trained using the full dataset, fine-tuning not reported	1.Program Structure and Content 2.Patient Engagement Metrics 3.Clinical Outcome Measures	Details about model validation not reported. The results are interpreted using graphs and text descriptions	Code available upon request, data publicly available (no database reported)
Ewbank et. al. 2020	UK	14,899 adult individuals (34.8±12.0 years old, 72.9% female, ethnicity not reported) who participated in eCBT for the treatment of a mental health disorder between June 2012 and March 2018	Association between the quality of each aspect of therapy and clinical outcomes using a deep learning approach	Various self-guided eCBT programs for psychiatric symptoms (6.2±2.9 weekly sessions)	Program dependent: 6.2 (SD=2.9) weeks	NA	Deep learning model	Yes	Model trained on 230/90,000 transcripts, and fine-tuned using 30/90,000 transcripts	24 therapy feature categories	Details about model validation not reported. The results are interpreted using graphs and text descriptions	Details about data or code availability not reported
Ewbank et. al. 2021	UK	34,000 adult individuals (median: 32 years old, 73.12% female, ethnicity not reported) who participated in eCBT for the treatment of a mental health disorder between June 2012 and October 2019	Association between patient utterances and clinical outcomes using a deep learning approach	Various self-guided eCBT programs for psychiatric symptoms (5.58±3.42 weekly sessions)	Program dependent: 5.58 (SD=3.42) weekly sessions	NA	Deep learning model	Yes	Model trained and tested on 270/340 and 20/340 randomly selected transcripts	5 patient response categories	Internal validation using 35/340 randomly selected transcripts (external validation not reported). The results are interpreted using graphs and text descriptions	Details about data or code availability not reported

NA, Not Applicable.

**Table 2:** Synthesis of data indices.

Variable	k	n	m	Participants	*τ^2^*	*I^2^*	*Fail-safe N*	*p*	Inconsistency analysis
Meta-analysis (pre vs post intervention analysis)									
Main effects	10	7	NA	840	0.1770 [0.0557; 0.7293]	71.3% [49.6%; 83.6%]	563	<0.0001	NA
Depression	7	4	NA	646	0.0361 [0.0000; 0.8054]	48.3% [0.0%; 75.9%]	200	<0.0001	NA
Anxiety	4	6	NA	185	0.000 [0.0000; 0.2786]	0.0% [0.0%; 74.6%]	6	0.0105	NA
Network meta-analysis (experimental vs control analysis)									
Main effects	6	8	36	462	0.1567 [0.0294; 0.8224]	65.5% [32.4%; 82.4%]	45	<0.0001	0.91
Depression	4	4	10	341	0.1248 [0.0108; 1.0364]	67.1% [21.8%; 86.2%]	53	0.0346	0.91
Anxiety	4	6	21	225	0.000 [0.0000; 0.2378]	0.0% [0.0%; 70.8%]	0	0.0928	NA
Dropouts	6	8	36	610	0.1123 [0.0000; 1.1416]	16.9% [0.0%; 58.0%]	0	0.0532	0.87

NA, Not Applicable.

### 3.3 Risk of bias within studies

Using the Cochrane Risk of Bias Tool and the additional risk of bias domains (selection, confounding, information, adherence, and allegiance bias), most studies were generally of moderate to low quality, presenting a high or unclear level of bias in multiple domains. Among the included studies, the domains that commonly presented a high risk of bias were allegiance bias (n=14) and selective reporting (n=8). In terms of allegiance bias, because of the scarcity and novelty of AI-augmented interventions and other AI algorithms, most of the included articles developed and studied their own AI tools, instead of AI tools developed by other researchers (29, 33, 73, 74, 76, 79, 80, 82–87, 90). The studies that ranked high for selective reporting bias tended to report only user satisfaction data and treatment adherence, despite their methods considering the collection and assessment of psychiatric scale data and other effectiveness outcomes (29, 76, 79, 85–88, 92). Other sources of bias, such as adherence, selection, confounding or information bias commonly presented a low risk of bias (**Supplementary Material 2**).

### 3.4 Effectiveness and tolerability of AI-augmented interventions for the management of mental health symptoms

#### 3.4.1 Main effects

**Figure 3:**
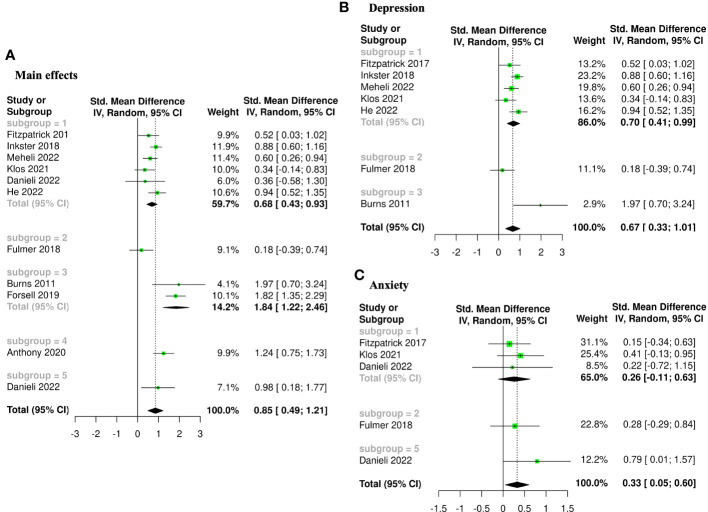
Forest plots of meta-analysis comparing pre- and post-intervention effects of five AI augmented interventions (Subgroup 1: AI self-guided eCBT, Subgroup 2: AI guided eCBT, Subgroup 3: AI modified eCBT, Subgroup 4: AI self-guided ACT, and Subgroup 5: Combined). **(A)** Main effects of all the included studies (n=10), **(B)** Interventions specific for the management of depression symptoms (n=7), **(C)** Interventions specific for the management of anxiety symptoms (n=4).

**Figure 4:**
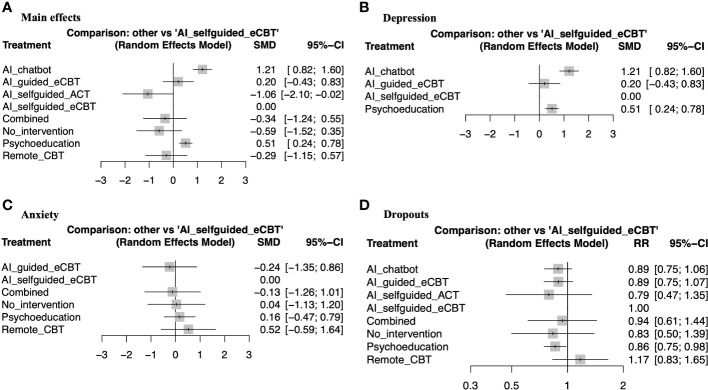
Forest plots of network meta-analysis of RCTs. **(A)** Main effects of all the included RCTs (n=6), **(B)** Interventions specific for the management of depression symptoms (n=4), **(C)** Interventions specific for the management of anxiety symptoms (n=4), **(D)** RCTs reporting participant dropouts (n=6).

The meta-analysis assessing the impact of AI-augmented interventions on mental health symptoms considered n=10 studies (41, 73–78, 80, 84, 90) presenting pre- and post-intervention outcomes. The results of this meta-analysis showed that these interventions had a significantly large effect size in the reduction of mental health symptoms. The sub-group analysis showed that AI-self-guided eCBT had a significant medium effect size, AI-guided eCBT effect had a non-significant effect, and AI-modified eCBT, AI-self-guided ACT and Combined had a significant large effect size in the reduction of mental health symptoms (**Figure 3A**). The NMA considered n=6 RCTs (41, 73, 74, 76–78). The head-to-head comparisons against AI-self-guided eCBT determined that this intervention was significantly more effective than AI chatbot with a large effect and psychoeducation with a medium effect size for the reduction of mental health symptoms. AI-self-guided ACT was significantly more effective than AI-self-guided eCBT with a large effect size for this outcome (**Figure 4A**). The head-to-head NMA ranked AI-self-guided ACT as the best treatment option for the reduction of mental health symptoms, with psychoeducation and AI chatbot being the lowest-ranked interventions (**Supplementary Material 7A**).

#### 3.4.2 Depression

The meta-analysis assessing the impact of AI-augmented interventions for the management of depression symptoms considered n=7 studies (74, 76–78, 80, 84, 90) presenting pre- and post-intervention depression score outcomes. The results of this meta-analysis showed that these interventions had a significant medium effect size in the reduction of depression symptoms. The sub-group analysis showed that AI-self-guided eCBT had a significant medium to large effect size, AI-guided eCBT effect had a non-significant effect, and AI-modified eCBT had a significant large effect size in the reduction of depression symptoms (**Figure 3B**). The NMA considered n=4 RCTs (74, 76–78). The head-to-head comparisons against AI-self-guided eCBT determined that this intervention was significantly more effective than AI chatbot with a large effect and psychoeducation with a medium effect size for the reduction of depression symptoms (**Figure 4B**). The head-to-head NMA ranked AI-self-guided eCBT as the best treatment option for the reduction of depression symptoms, with psychoeducation and AI chatbot being the lowest-ranked interventions (**Supplementary Material 7B**).

#### 3.4.3 Anxiety

The meta-analysis assessing the impact of AI-augmented interventions for the management of anxiety symptoms considered n=4 studies (73, 74, 76, 78)presenting pre- and post-intervention anxiety score outcomes. The results of this meta-analysis showed that these interventions had a significant small effect size on the reduction of anxiety symptoms. The sub-group analysis showed that AI-self-guided eCBT had a significant small effect size, AI-guided eCBT effect had a non-significant effect, and AI-modified eCBT had a significant medium to large effect size in the reduction of anxiety symptoms (**Figure 3C**). The NMA considered n=4 RCTs (73, 74, 76, 78). The head-to-head comparisons against AI-self-guided eCBT determined that the effects of this intervention were not significantly different than AI-guided eCBT, combined, no intervention, psychoeducation, and remote CBT (**Figure 4C**). The head-to-head NMA ranked AI-guided eCBT as the best treatment option for the reduction of anxiety symptoms, with psychoeducation and remote CBT being the lowest-ranked interventions, though none of these comparisons were statistically significant (**Supplementary Material 7C**).

#### 3.4.4 Adverse events and attrition rate

None of the included studies reported adverse events associated with the interventions. 12 of the included studies (41, 73–83) presented data on treatment dropout, online or remote psychotherapy interventions reported a dropout rate between 0% and 61%, psychoeducation a dropout rate between 4% and 60% and no intervention a dropout rate between 5% and 34%. No specific trends were identified when comparing the attrition rates with intervention length or whether the online intervention was therapist guided or not (**Table 1**). The NMA considered n=6 RCTs (41, 73, 74, 76–78). The head-to-head comparisons against AI-self-guided eCBT determined that this intervention had a 14% lesser risk of dropping out compared to psychoeducation, this comparison was statistically significant. The other head-to-head comparisons against AI chatbot, AI-guided eCBT, AI-self-guided ACT, Combined and Remote CBT were not statistically significant (**Figure 4D**). The head-to-head NMA ranked remote CBT as the best treatment option in terms of lower attrition rates, with psychoeducation and AI-self-guided ACT being the lowest-ranked interventions (**Supplementary Material 7D**).

#### 3.4.5 Limitations in the assessment of effectiveness and tolerability

An important limitation of this meta and network meta-analysis is the scarcity of studies in this field. Aside from AI-self-guided eCBT [n=6 (73, 74, 77, 78, 84, 90)] and psychoeducation [n=4 (74, 76–78)], other interventions and comparators were reported in three or fewer studies – AI-modified eCBT [n=3 (75, 79, 80)], no intervention [n=2 (41, 73)], AI-guided eCBT [n=1 (76)], AI-self-guided ACT [n=1 (41)], AI chatbot [n=1 (77)], combined [n=1 (73)], remote CBT [n=1 (73)]. This can increase the imprecision, the indirectness, and the impact of bias in the results obtained with these analyses (54). **Supplementary Materials 3**-**6** shows the full forest plots of the NMA analysis, for main effects, depression, anxiety, and dropout-related data respectively.

#### 3.4.6 AI treatment user satisfaction and tool usage

In terms of user satisfaction with AI-delivered online psychotherapy tools, several studies (n=10) reported that their study tool was well received and perceived as helpful and encouraging by around 60% to 90% of users. These studies highlighted the number of interactions with the tool, a sense of empathy and understanding, and the appropriateness of the dialogue as important positive factors determining treatment outcomes and satisfaction. They also reported that a minority of users, around 30% or less, thought that the tool did not understand them or found their interactions unhelpful or bothersome (73, 74, 76–78, 80–82, 84, 90).

In terms of tool usage and outcomes, Anthony et al. (2020) found that their AI-delivered online ACT intervention for postoperative pain, resulted in users consuming 36.5% fewer opioid tablets than the control group and significantly lower postoperative pain (41). Binik et al. (1988) reported that users of their AI-delivered virtual sexual dysfunction therapy tool (Sexpert) appeared comfortable discussing issues with their sexuality and other intimate topics with the tool (82). Danieli et al. (2022) found that when added to traditional in-person CBT, their AI-delivered online psychotherapy tool (TEO), promoted a greater improvement in stress levels and overall well-being compared to either in-person CBT or TEO by itself (73). Frias et al. (2021) determined that younger patients, those with a higher level of emotional dysregulation, and those with a higher level of education reported a high level of tool (B·RIGHT) usability (81).

### 3.5 AI improvements and feature detection algorithms

AI technology has been used to find ways to improve the features or the delivery of previously validated online psychotherapy treatments. In terms of improvement to the delivery of online therapy, the included studies [n=4 (30, 75, 79, 80)] have reported on the implementation of intelligent notifications, momentary sensing, monitoring of treatment outcomes, and triage decision support. Burns et al. (2011) implemented an AI momentary sensing tool (Mobilize) which used mobile device sensor data, to determine the appropriate time to deliver a treatment notification based on a participant’s mood and location. This tool demonstrated a 60% to 91% accuracy in location prediction (no significant results in predicting mood state) and significant effectiveness improving symptoms of depression (80). Morrison et al. (2017) implemented a similar momentary sensing approach to develop intelligent notifications to support treatment engagement. They found that compared to occasional notifications, intelligent notifications and daily notifications encouraged a higher exposure to the therapeutic content while maintaining engagement levels. However, they noted that the timing of the notification did not seem to support treatment engagement, when comparing intelligent notifications against daily notifications (79). Gonzalez Salas Duhne et al. (2022), implemented an AI algorithm which used demographic and clinical factors to determine whether participants would benefit from face-to-face guided self-help or eCBT retrospectively. Participants who received the appropriate intervention determined by AI experienced improved treatment outcomes (OR=2.10) and a lower risk of dropping out (OR=1.12) compared to the other participants. Notably this study reported that despite its availability, 98% of the sample received face-to-face therapy, while according to their AI algorithm 96% of participants with mild to moderate depression symptoms would have benefited from eCBT (30). Forsell et al. (2019) implemented an AI monitoring tool to detect participants at risk of dropping out of their eCBT program for insomnia. At-risk participants were randomized to either continue with their program or receive an adapted version including additional therapist interaction (around 14 minutes per week). This adaptation supported higher symptomatic improvement and a lower risk of dropping out (OR=0.33), compared to at-risk participants that continued with their standard eCBT program (75).

In terms of feature detection algorithms to improve online therapy delivery, the relevant studies [n=3 (85, 86, 91)] focused on the content of the therapy program and the utterances of the participants and found several factors that could promote and predict higher effectiveness and engagement. Ewbank et al. (2020) and Ewbank et al. (2021) applied a deep learning approach to label large datasets of patients and therapy transcripts obtained from a variety of eCBT programs for mental health symptoms (85, 86). Ewbank et al. (2020) determined that for therapy structure, the time spent on cognitive and behavioral techniques (changed methods content) was associated with higher odds of symptomatic improvement (OR=1.11) and treatment engagement (OR=1.12) compared to non-therapy-related content. Though they recognize that some non-therapy-related content such as greetings can be important for the session, excessive amounts of it can be distracting and ultimately detrimental. They also reported that the model had a precision of 50% to 100%, and human-level accuracy when labeling therapy transcripts (86). Ewbank et al. (2021) found that patients’ statements showing a desire or commitment to change (Change-talk active: OR=1.38 and Change-talk explore: OR=1.14) were associated with increased odds of symptomatic improvement and engagement. Comparatively, statements that moved away from the target behavior (Counter-change talk: OR=0.80) or that were not related to change (Neutral/follow: OR=0.94), were associated with poorer symptomatic improvement and adherence. They also reported that the model had human-level accuracy when labelling therapy transcripts, with a precision of around 50% to 80% for most labels (human assessor was better at labelling “Counter-Change talk”: 62% vs 18% than the model, and the model was better at labelling “Describing problems”: 57% vs 22% than the human assessor) (91). Myllari et al. (2022) applied a text-mining approach to identify common topics in the written assignments of patients participating in an eCBT program for generalized anxiety disorder. They reported that patients who wrote about the well-being of their family and loved ones showed faster and better symptomatic improvement, compared to those who wrote about monitoring thoughts, worries, and concerns about internet therapy (91).

### 3.6 AI prediction algorithms

About half of the identified studies (41.4%) focused on the implementation and analysis of a variety of AI prediction algorithms for different outcomes in online mental healthcare, including treatment responses and symptom remission, treatment dropouts, and treatment engagement and adherence (28–32, 87–89, 91–94). The identified studies applied ML algorithms such as: DL, and ANN algorithms to process large amounts of treatment data to identify helpful prediction factors. The specific AI data analysis approaches used by the relevant studies and an assessment of study elements following research guidelines and standards for ML studies (i.e., adequacy of the data for the intended outcomes, model training and fine-tuning, features analyzed, validation, interpretability, and code and data availability) are presented in **Table 1**. All ML studies [n=15 (28–33, 85–89, 91–94)] presented a comprehensive description of their data, justification for the implementation of ML in their analysis, and a description of the features analyzed. 80% of studies reported the data or approach used to train their ML algorithms (28–30, 32, 33, 85–89, 92, 93), 53.33% of studies reported the use of model fine-tuning (28, 30, 32, 85–87, 91, 93, 94), 73.33% of studies reported the use of internal validation methods (28–32, 85, 88, 89, 92–94), 13.33% of studies reported the use of external validation methods (28, 30), 26.67% of studies reported that the data or the code used in the study can be made available upon reasonable request (28, 30, 33, 92, 93), 20% of studies included a link to the repository for their ML algorithm code (29, 88, 93), and 6.67% of studies included a link to the repository for the dataset used (87).

#### 3.6.1 AI treatment response prediction

For treatment response prediction, the relevant studies [n=9 (28, 29, 31–33, 88, 89, 92, 93)] focused on the identification of demographic and clinical factors [n=8 (28, 29, 31–33, 88, 89, 92, 93)], and engagement styles [(33)]. The 8 studies that focused on demographic and clinical factors included: Flygare et al. (2020) identified depressive symptoms, treatment credibility, number of body areas of concern, duration of symptoms, and working alliance as predictors of symptom remission with 78% accuracy, in patients participating in an eCBT program for BDD. They noted that the level of BDD insight and demographic variables were not important predictors of remission, explaining that treatment response may be more directly influenced by treatment factors (29). Lenhard et al. (2018) identified younger age of symptom onset, and duration and severity of symptoms in patients participating in eCBT for OCD were predictors of poorer treatment response with 75% to 83% accuracy. Based on these results they suggested that eCBT for OCD may be better suited for individuals with mild to moderate symptoms (88). Mansson et al. (2015) used fMRI and a machine learning approach to identify patterns of brain activation associated with long-term treatment response. They identified that patterns of Blood-Oxygen-Level Dependent response in the dorsal part of the anterior cingulate cortex and the amygdala could predict treatment response with 92% accuracy 1 year after participation in an eCBT program for social anxiety disorder (89). Pearson et al. (2019) identified pre-treatment assessments, comorbid psychopathology, disability, treatment credibility, and module usage as predictors of treatment response in patients receiving online psychotherapy for depression (32). Rocha et al. (2018) found that an average of 66% predicate engagement (messages exchanged with the platform) was positively associated with treatment outcomes, and that adherence to the treatment platform (eCBT for depression) predicted treatment outcomes with about 60% accuracy. Because of the low prediction accuracy, they explained that treatment engagement alone may not be a strong predictor of clinical improvement (31). Rodrigo et al. (2021) and Rodrigo et al. (2022) applied different AI approaches to identify predictors of treatment response in an eCBT program for tinnitus (92, 93). Rodrigo et al. (2021) applied decision trees models and determined that education level (master’s degree or higher education level 88% chance of treatment success), baseline tinnitus severity, and depression and anxiety symptoms were predictors of treatment response with around 70% accuracy (92). Rodrigo et al. (2022) applied an ANN and machine learning approach and determined that higher education level, older age, employment status (i.e., no or fewer work restrictions), baseline tinnitus severity, and lower insomnia scores were predictors of treatment response with 78% accuracy. These studies noted that additional factors may be at play determining a lower response success for individuals with a lower education background and argued for more resources targeted at this patient population (93). Wallert et al. (2022) reported that polygenic risk factors for intelligence was the most important genetic predictor, pre-treatment MADRS-S was the most important clinical predictor, and the time of day when the patient completed their MADRS-S was the most important treatment process predictor of symptom remission after participation in an eCBT program for depression with around 65% accuracy. They recognized that this accuracy level did not outperform other models in the literature (28).

One study focused on engagement styles: Chien et al. (2020) classified the participants of an eCBT program for depression into 5 classes of treatment engagement which considered treatment platform usage (i.e., time spent on the platform, access to therapy sessions and tools, and therapy session completion) and rate of treatment disengagement. They found that lower platform usage was associated with a lower rate of symptom improvement and that higher platform usage and low rate of disengagement were associated with higher symptom reduction for depression and anxiety symptoms. However, they noted that this classification did not consider the impact of sociodemographic factors because the used dataset only contained de-identified information (33).

#### 3.6.2 AI treatment dropout prediction

For treatment dropout prediction [n=2 (30, 83)], Bremer et al. (2020) focused on the analysis of user journeys and identified that time spent in earlier stages of the program, morning wake-up times (either before 4:30 am or later than 6:45 am), time to get out of bed (less than 9 minutes or more than 66 minutes), a greater wake after sleep onset, logging triggers for 18 days or more, receiving emails for 30 days or more, and no interaction with the treatment platform for over 67 days could predict dropout early in the treatment (eCBT for insomnia) with 60% to 90% accuracy (83). Gonzalez Salas Duhne et al. (2022), implemented a supervised ML approach to analyze data from a face-to-face and an online CBT program for depression, and identified five common variables that could predict a higher probably of early dropout from online CBT: younger age, belonging to an ethnic minority, lower socioeconomic status, medications, and higher baseline severity of depression symptoms (30).

#### 3.6.3 AI treatment engagement and adherence prediction

For treatment engagement and adherence prediction [n=2 (87, 94)], Kim et al. (2021) determined that higher engagement in an eCBT program for obesity was associated with higher weight loss. The implemented machine learning approach identified self-esteem, in-app motivational measures, lower intake of high-calorie food, and higher interaction frequency with a healthcare mentor as predictors of higher treatment engagement. Because of the association found between treatment engagement and response, similar factors predicted short-term and long-term weight loss, with lower lunch and evening snack intake, lower fat intake, lower step count, higher will and higher confidence being additional predictors for long-term weight loss. They reported that around 59% of outcome variance was explained by their prediction model (87). Wallert et al. (2018) reported that for patients participating in an eCBT program for post-myocardial infarction depression and anxiety, self-assessed cardiac-related fear, female sex, number of words used to complete the first homework, and self-rated depression were predictors of adherence with 64% accuracy. They noted that contrary to the literature, education level and age did not show a strong predictive power in this study (94).

### 3.7 Risk of bias across studies and quality of the evidence

Analysis of heterogeneity showed that for the meta-analysis and the NMA, the main effects outcome had the highest degree of heterogeneity (I^2^>50%). In the NMA, the depression outcome also had a significant degree of heterogeneity (I^2^>50%) (**Table 2**) (43, 59–62). Analysis of funnel plots (**Supplementary Material 8**) and Rosenthal’s fail-safe N (**Table 2**) showed that most reported outcomes presented a low degree of publication bias (p<0.05), except for anxiety and dropouts in the NMA (55). Analysis of inconsistency showed that the NMA model had a good fit for the analysis of study outcomes (p>0.05) (54) (**Table 2**). Analysis of imprecision showed that for main effects, the analysis model had a low level of imprecision when comparing AI-self-guided eCBT to AI chatbot, psychoeducation and AI-self-guided ACT. For depression, the analysis model had a low level of imprecision when comparing AI-self-guided eCBT to AI chatbot and psychoeducation. For anxiety all comparisons had a high level of imprecision, and for treatment dropout data, only the comparison between AI-self-guided eCBT and psychoeducation had a low level of imprecision (**Figure 4**) (54, 57, 58). Analyzing indirectness, shows that most head-to-head comparisons arose from indirect data comparisons, rather than comparisons presented in the literature (direct comparisons) (54) (**Supplementary Material 7**). In terms of risk of bias, most studies presented a high level of bias in several of the assessed domains (**Supplementary Material 2**). Therefore, applying the GRADE guidelines (54) the quality of the data was low to very low.

## 4 Discussion

This mixed-methods review aimed to present a comprehensive assessment of the current uses of AI in online mental healthcare using meta-analytic and network meta-analytic approaches. Our systematic search strategy identified various applications including triage, psychotherapy delivery, therapy progress monitoring, therapy engagement support, identification of engagement subtypes, effective therapy features and themes in patients’ utterances, and prediction of short-term and long-term treatment response, treatment dropout and adherence, and symptom remission (28–33, 41, 73–94). Analyzing these applications, the results of this review suggested that despite the novelty of this technology, AI has already demonstrated promising benefits to the field of online mental healthcare. The meta-analysis of the AI-augmented interventions suggested positive beneficial results in the management of mental health symptoms, and the NMA ranked AI-augmented interventions including eCBT and ACT as the best interventions for the management of mental health symptoms compared to other interventions (**Supplementary Material 7**). Additionally, AI-augmented interventions were well tolerated, with no study reporting side effects from the use of these interventions and AI-self-guided eCBT being among the top-ranked interventions by the NMA head-to-head analysis in terms of lower attrition rate. However, it is important to note that the quality of the evidence was low to very low according to the GRADE criteria (54). The quality of the data was mainly impacted by the scarcity of studies in this field, the significant presence of allegiance bias, and the heterogeneity of the studies. Therefore, the results of this review should be considered and interpreted with caution.

The available literature supports the results obtained by this mixed-methods review, showing that AI-augmented interventions such as AI therapy chatbots can be promising, effective and accessible solutions for mental healthcare delivery (6, 17–23). For instance, two recent reviews by Boucher et al. (2021) and Abd-alrazaq et al. (2019), reported that chatbots had shown promising applications including care assistance, symptom screening and monitoring, and therapy delivery. These reviews recognized the novelty of this technology and the scarcity of evidence but noted several potential benefits in terms of providing a low-resource intensity alternative for individuals experiencing less severe mental health symptoms (6, 17). This in turn could help reduce the burden on the healthcare system and help direct individuals with more severe symptoms to the appropriate in-person services. Additionally, this review suggested that the general population seems interested in AI-augmented interventions, presenting high satisfaction and generally positive perceptions of this technology (6, 17). Another review by Graham et al. (2019) also emphasized the infancy of the evidence but noted several favorable applications of AI technology. This review noted that this technology has demonstrated high accuracy in symptom detection and prediction, and early disease detection due to its ability to analyze and interpret large data sets (20). Likewise, the results of this review encourage a cautious approach when interpreting the available literature, while highlighting potential areas where AI could enhance or support online mental healthcare.

Considering the social and economic burden presented by mental health illness, and that worldwide approximately 70% of people receive no formal treatment for their mental health symptoms, there is a critical need for increased service accessibility. These service gaps were exacerbated by the limitations of the COVID-19 pandemic, highlighting the importance of accessible mental health services, such as online mental healthcare interventions (6, 17). The various AI-augmented applications presented in this review have achieved encouraging positive mental health outcomes, while leveraging and supporting the benefits of online platforms, including scalability, versatility, cost-effectiveness, and personalization (8, 9, 95–97). Moreover, despite the accessibility of online mental healthcare interventions, reduced therapist support or guidance in some online programs, have resulted in participants having low levels of engagement with their treatment and a high risk of dropping out (6). AI-augmented interventions could help address this problem, while maintaining a low level of resource utilization (no or reduced therapist involvement) through the use of human-like communication, intelligent treatment reminders, environment sensing to deliver more accurate treatment engagement support, and symptom monitoring (17, 20, 22, 23). For instance, the therapeutic AI agents or chatbots identified by our systematic search (i.e., Wysa, Woebot, TEO, Tess, XiaoE, and Sexpert) were effective at delivering self-guided therapy content by communicating intelligently with their users, promoting a significant reduction in mental health symptom severity with high user satisfaction (73, 74, 76–78, 82, 84, 90). For instance, Fitzpatrick et al. (2017) stated that participants in their study reported that the bot (Woebot) felt empathetic and commented on it as “a friend” and a “fun little dude” (74). This presents the interesting possibility of a potential therapeutic relationship between user and chatbot, which is something that self-guided eCBT has not been able to accomplish (6, 17–23, 74). In terms of treatment engagement support, Burns et al. (2011) and Morrison et al. (2017) implemented an AI algorithm that could learn from the user and their environment, to deliver intelligent treatment reminders (79, 80). Though, in the case of Morrison et al. (2017) they found that there was no significant improvement comparing daily to intelligent notifications (79, 80).

Additionally, the studies reporting on the use of ML algorithms found significant success in outcome prediction and feature detection to support online therapy delivery (6, 19, 28, 29, 31, 33, 83, 85, 86, 92–94). This function of AI technology could promote significant breakthroughs in the design, development, and delivery of online mental healthcare by supporting the inclusion of helpful therapy features, identifying patients who may need higher mental health support, and modifying therapy delivery to support better outcomes and adherence. For instance, the study by Gonzalez Salas Duhne et al. (2022) reported that their ML algorithm successfully suggested eCBT as the most appropriate treatment for 96% of analyzed patients, finding improved treatment response and adherence for the patients who matched this recommendation. However, because of patient and practitioner factors (i.e., preference, lack of familiarity with eCBT, etc.), despite availability and accessibility of eCBT and longer wait-times for face-to-face interventions, 98% of the analyzed patients received the face-to-face option instead of eCBT (30). This result emphasizes AI technology’s impact on clinical practice and decision-making. As such, the relevant studies highlighted the importance of analyzing the large datasets produced by online mental healthcare interventions and the efficiency and accuracy of AI algorithms which in some cases were comparable to or better than human assessors (6, 19, 28, 29, 31, 33, 83, 85, 86, 92–94). Another application of this technology was presented in a review by Fonseka et al. (2019), which reported over 80% accuracy in suicide risk prediction achieved by AI models, by considering the complex interactions of psychosocial, social, biological, and environmental factors. They also reported that this predictive power could be enhanced through the interface of AI with the Internet of Things (IoT) to capture comprehensive biometrics from a person’s daily life (19).

However, despite the potential benefits of AI technology for the advancement of online mental healthcare, there are still several unanswered questions and concerns requiring further research. With the advancement of online mental healthcare, care providers and participants alike have commented on the potential detrimental reduction of therapeutic face-to-face interaction and its impact on the therapeutic alliance (8, 24, 46). Likewise, with the advancement of AI-delivered online mental healthcare, there has been a concern over losing a human element to therapy and the value of empathetic care. In a global survey of psychiatrists, around 83% of respondents felt that AI would not be able to match the level of empathetic care provided by a professional (6, 98). Some users have reported that they are likely to share less information with a chatbot, compared to a human counsellor (18). This could limit the effectiveness of AI-augmented interventions, creating potentially risky situations in which patients may avoid discussing important topics regarding their mental health. Moreover, AI chatbots and language models have been inefficient at interpreting and responding to idioms, colloquialisms, slang, and metaphors. This limitation can be frustrating for users if they feel that the AI service does not understand them or their needs, making the interaction feel less human-like impacting treatment adherence, and may impact the prediction abilities and accuracy of large language models (6, 17, 18, 99). In this regard, the study by Inkster et al. (2018) reported that around 32% of participants did not find their interactions with the AI chatbot helpful, and in some cases found their interactions bothersome or annoying (6, 84). Additionally, the scarcity of studies and low quality of evidence, including the lack of a control or adequate placebo group impacts the strength and reliability of the reported outcomes (6, 17, 18). In terms of large data analysis using ML algorithms, if not properly accounted for, several factors can impact the accuracy and reliability of the results. (34–37) Model fitting issues for instance can result in unapplicable or unreliable models that either rely too closely on the training data and perform poorly when they encounter new data (overfitting), or that do not have sufficient training data to make meaningful predictions (underfitting). For instance, DL consider millions of training parameters, as such insufficient training data can result in a model that has a high accuracy for the training data but poor generalizability outside of the study (34–37). This may be an important issue considering the novelty and sometimes scarcity of online mental healthcare data, compared to traditional face-to-face data (6, 17–23). Data leakage which can result from improper data handling during model training can be hard to detect and may significantly impact the applicability of the model’s results to the real-world. Another issue is the fine-tuning of model hyperparameters to support model accuracy and interpretability. In ML, the higher the accuracy, the higher the model complexity and the lower the interpretability of the models results become for a human assessor. In this review, most of the included articles did not follow the guidelines and standards for ML studies, for instance most studies did not report on their use of fine-tuning, did not present external validation considerations or results, and did not make their algorithm code publicly available (34–37). These factors should encourage the readers to exercise caution when interpreting the results of studies implementing ML algorithms (34–37). There are also ethical concerns regarding the use of AI technology for the delivery of therapy. Since the accuracy of AI technology relies on the reliability and accuracy of the data used for model training, any biases present in the data can adversely affect the model’s usability in unpredictable and potentially harmful ways. For instance, linking mental illness to certain ethnicities or genders, providing inaccurate mental health information and encouraging risky behaviors (6, 20). In this regard, several studies included in this review which aimed to predict treatment response, identified demographic characteristics often related to more affluent and less marginalized populations as predictive of better treatment response, showcasing this potential bias in the data. As an example, the two studies by Rodrigo et al. (2021 & 2022) identified higher education level (master’s degree and above) as a strong predictor of treatment response applying a DL approach, noting that this result may also underly additional factors in the way that the studied treatments were delivered and accessed (92, 93). Similarly, Wallert et al. (2018) noted that education level is a commonly reported predictive factor of treatment response, in the literature regarding patients participating in eCBT (94). Finally, healthcare policy may not be able to catch up fast enough to the quick advancement and implementation of AI technology, which may create some unexpected consequences in clinical practice (6, 17–23). As such, prioritizing the principles of autonomy, beneficence, and justice, and addressing technology literacy gaps amongst users is crucial in this process (20). This will ensure that the needs and wellbeing of patients are protected and upheld, while supporting the evolution of this transformative and promising technology.

In conclusion, the results of this review suggest that AI-augmented interventions and tools are promising additions to online mental healthcare. Our results supported the effectiveness and tolerability of AI-augmented interventions in the reduction of mental health symptom severity, with high user satisfaction and attrition rates similar or lower compared to other interventions in the field. These are encouraging results that aim to advance the development and implementation of effective and accessible online mental healthcare interventions in clinical practice. For instance, AI services can support treatment engagement and monitoring, improving the effectiveness of online mental healthcare interventions and treatment adherence. Furthermore, our review highlighted the efficiency and accuracy of ML algorithms, which has been shown to be comparable or better than humans assessors for a variety of tasks (i.e., treatment outcome predictions, feature identification, treatment adherence and engagement prediction). Nevertheless, the novelty of this field and the quality of the available data directly impact our ability to interpret these results and emphasize the need for more adequately powered high-quality RCTs exploring the effects of AI-augmented online mental healthcare interventions.

### 4.1 Strengths and limitations

The results of this review synthesized the available data to support the understanding of this novel and rapidly evolving technology. The design of this study had several strengths including the use of validated evidence-based analytical methods for study search and selection, and data extraction and synthesis, following PRISMA guidelines and the extension for NMAs (42–44). Additionally, we used a rigorous approach for risk of bias and data quality assessment applying the Cochrane risk of bias tool and the GRADE guidelines respectively. However, though the mixed methods review approach allows for an in-depth analysis of the available evidence, there are important limitations worth noting when interpreting these results. First, the quality of the available data is impacted by the scarcity of studies, the level of indirectness, the high risk of bias, and the heterogeneity of the studies. The scarcity of studies and novelty of the field also influenced the impact of allegiance bias and the heterogeneity of the evidence. For instance, the data related to AI-guided eCBT, AI-self-guided ACT, combined and remote CBT, AI prediction of treatment dropout, AI identification of useful therapy aspects, AI identification of predictors of symptom remission, AI matching patients to appropriate treatments, AI prediction of treatment adherence, and AI prediction of symptom remission were extracted from just one study. In addition, without a defined standard or guidelines for the study and implementation of AI tools in online mental healthcare, most research teams opted for the study and development of their own AI tools, comparator interventions, outcome metrics, and intervention designs (43, 59–62). Moreover as mentioned by other reviews in the field, several of the available studies lack an appropriate comparator or control group which can directly impact the observed effect of the AI-augmented interventions and tools reported in the literature (6, 17–23, 52–54, 57, 58, 67, 72). Second, the “main effects” outcome analysis combined the results of studies with different designs and outcome measures. This approach accounted for the scarcity of the data and allowed us to make a general assessment of intervention effects for the management of non-specific mental health symptoms. However, the results of the main effects outcome should be interpreted with caution accounting for the impact of heterogeneity and low data quality (54). Third, though no adverse events were identified during the data extraction step, it is worth noting that there is a recognized trend of underreporting this type of data in online mental healthcare studies. For instance, a study by Sundström et al. (2020) which acknowledges this gap in the data, identified several side effects associated with the implementation of an eCBT program for alcohol use disorder, including increased cravings for alcohol, increased consumption of alcohol and feelings of distress related to the intervention (100). Therefore, the assessment of the tolerability of AI-augmented interventions should consider the potential of side effects underreporting. Finally, it is worth noting the factors that may impact the generalizability of the results. This includes gender distribution, with most studies (n=20) including >50% female participants; the ethnicity distribution in the included studies, with most studies being conducted in the USA (n=10), Sweden (n=6), and the UK (n=6), and including a populating that predominantly identified as white; and the exclusion of studies not written in English, which may have limited the scope of our review to Western societies.

### 4.2 Future directions

The results of the study supported the promising impact of AI-augmented intervention and tools; however, the scarcity and quality of the available data highlighted the need for more adequately powered high-quality RCTs. Furthermore, the direct and indirect NMA comparisons laid out potential next steps to help validate the effectiveness of this technology. For instance, the NMA results identified AI-self-guided ACT as a more effective intervention than AI-self-guided eCBT at alleviating mental health symptoms. However, this result stemmed from an indirect comparison, and only one study reported on the effects of AI-self-guided ACT impacting the power of this result. Other direct and indirect comparison results also included interventions presented in only one study, such as: AI-guided eCBT, and combined AI-self-guided eCBT and remote CBT. Thus, future studies could focus on these results to guide the development and validation of AI-augmented interventions. Moreover, to support the development of this technology and the robustness of future studies, it is important to consider the possibility of developing large open-source datasets in mental health which could serve as benchmarks for AI algorithm development. Additionally, it is important to acknowledge that the implementation and use of these novel and promising interventions may be restricted to those with a reliable level of technology literacy, and access to the internet and internet-enabled devices (95, 101). Hence, though online mental healthcare is intended to promote higher service accessibility, especially for those living far away from in-person services (8, 24), these systemic barriers can limit the intended benefit of these interventions. Future studies should acknowledge this factor and support service accessibility by lending internet-enabled devices or supporting technology literacy for marginalized communities (8, 9, 95, 102).

## Data availability statement

The raw data supporting the conclusions of this article will be made available by the authors, without undue reservation.

## Author contributions

GG: Conceptualization, Data curation, Formal Analysis, Investigation, Methodology, Project administration, Software, Supervision, Validation, Visualization, Writing – original draft, Writing – review & editing. CS: Data curation, Formal Analysis, Investigation, Methodology, Writing – original draft, Writing – review & editing. JE: Conceptualization, Data curation, Investigation, Methodology, Visualization, Writing – original draft, Writing – review & editing. KA: Conceptualization, Data curation, Investigation, Methodology, Visualization, Writing – original draft, Writing – review & editing. NA: Conceptualization, Methodology, Project administration, Supervision, Validation, Writing – original draft, Writing – review & editing.
