# Supplementary material for: Examining the role of AI technology in online mental healthcare: opportunities, challenges, and implications, a mixed-methods review
Source: Front Psychiatry. 2024 May 7;15:1356773. doi: 10.3389/fpsyt.2024.1356773 (PMC11106393; doi:10.3389/fpsyt.2024.1356773)

Supplementary Material

**Supplementary data 1:** Search strategy

(“Artificial intelligence” [MeSH Terms] OR “AI” OR “Algorithms” [MeSH Terms] OR “Machine learning” [MeSH Terms] OR “NLP” OR “Natural language processing” [MeSH Terms] OR “Neural network, computer” [MeSH Terms] OR “deep learning” [MeSH Terms] OR “AI technology”) **AND** (“clinical decision-making” [MeSH Terms] OR “Decision making” [MeSH Terms] OR “Diagnosis, Computer-assisted” [MeSH Terms] OR “stepped care” OR “clinical monitoring” OR “Monitoring, physiologic” [MeSH Terms] OR “personalized care” OR “symptom monitoring” OR “Diagnosis, Differential” [MeSH Terms] OR “diagnosis” [MeSH Terms] OR “symptom detection” OR “prognosis” [MeSH Terms] OR “clinical outcomes” OR “treatment outcome” [MeSH Terms] OR “triage” [MeSH Terms] OR “General practice” [MeSH Terms] OR “treatment stratification” OR “therapy delivery” OR “Clinical competence” [MeSH Terms]) **AND** ("relapse prevention” OR “relapse prevention therapy” OR “RPT” OR “cognitive behavioral therapy” [MeSH Terms] OR “dialectic behavioral therapy” OR “DBT” OR “counselling” [MeSH Terms] OR “talk therapy” OR “CBT” OR “behaviour therapy” [MeSH Terms] OR “impulse control” OR “cognitive therapy” OR “psychotherapy” [MeSH Terms] OR "behavior modification” OR "psychoeducation" OR “mindfulness” [MeSH Terms] OR “online psychotherapy” OR “electronic psychotherapy” OR “asynchronous psychotherapy” OR “therapy, computer-assisted” [MeSH Terms] OR “online CBT” OR “online DBT” OR “online RPT”) **AND** ("therapy software" OR "web-based intervention" OR "internet" [MeSH Terms] OR "internet-based intervention" [MeSH Terms] OR "online" OR "electronics" [MeSH Terms] OR "software" [MeSH Terms] OR "online platform” OR "online modules" OR "distance education" [MeSH Terms] OR "e-learning” OR “virtual education” OR “computer-assisted instruction” OR “technology” [MeSH Terms])


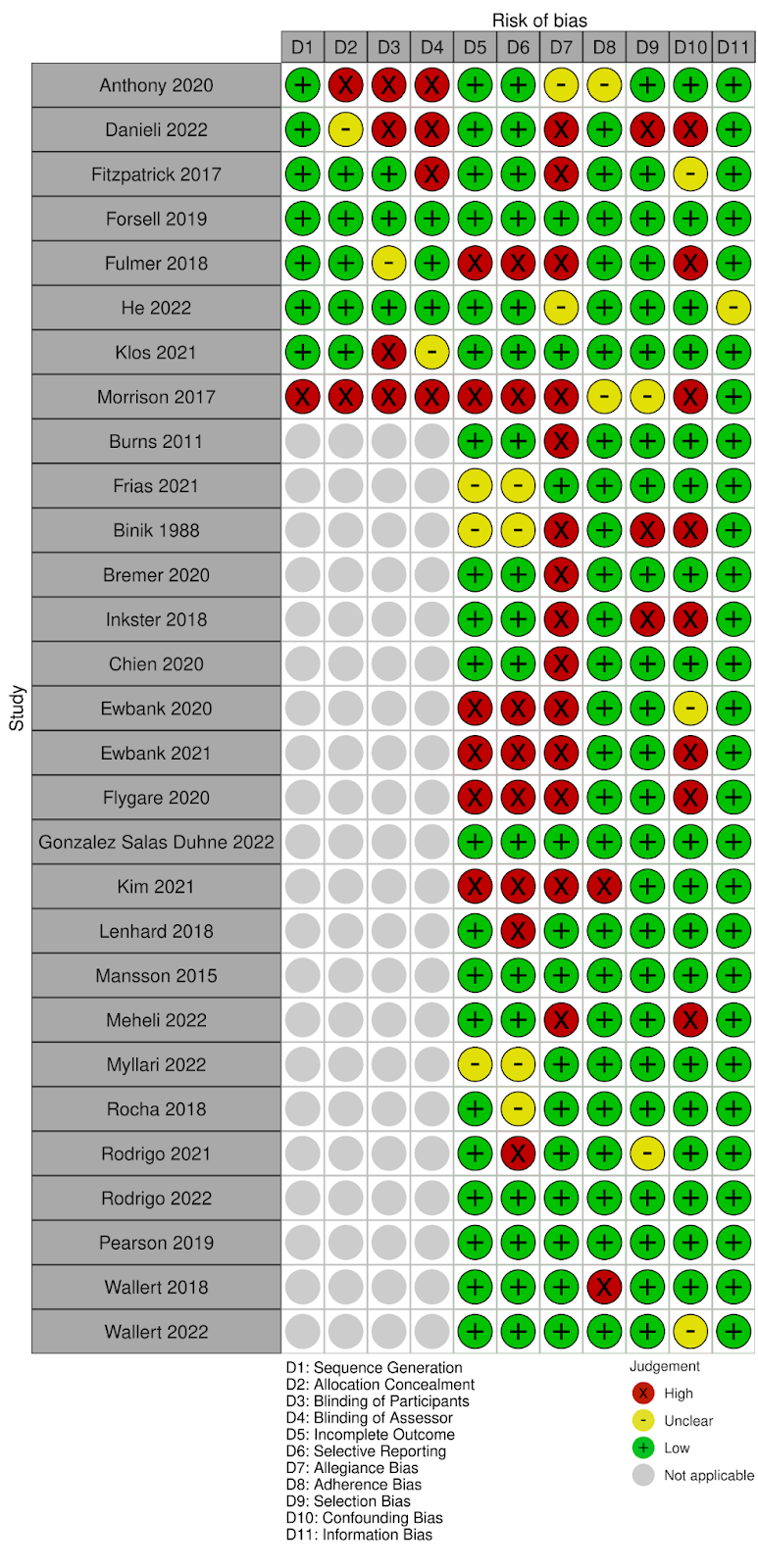
**Supplementary data 2:** Risk of bias, showing the assessment (high = red, unclear = yellow and low= green) of 11 risk of bias domains.

**Supplementary data 3:** Forest plots using a random effects model considering the main effects of all the included RCTs (n=6)


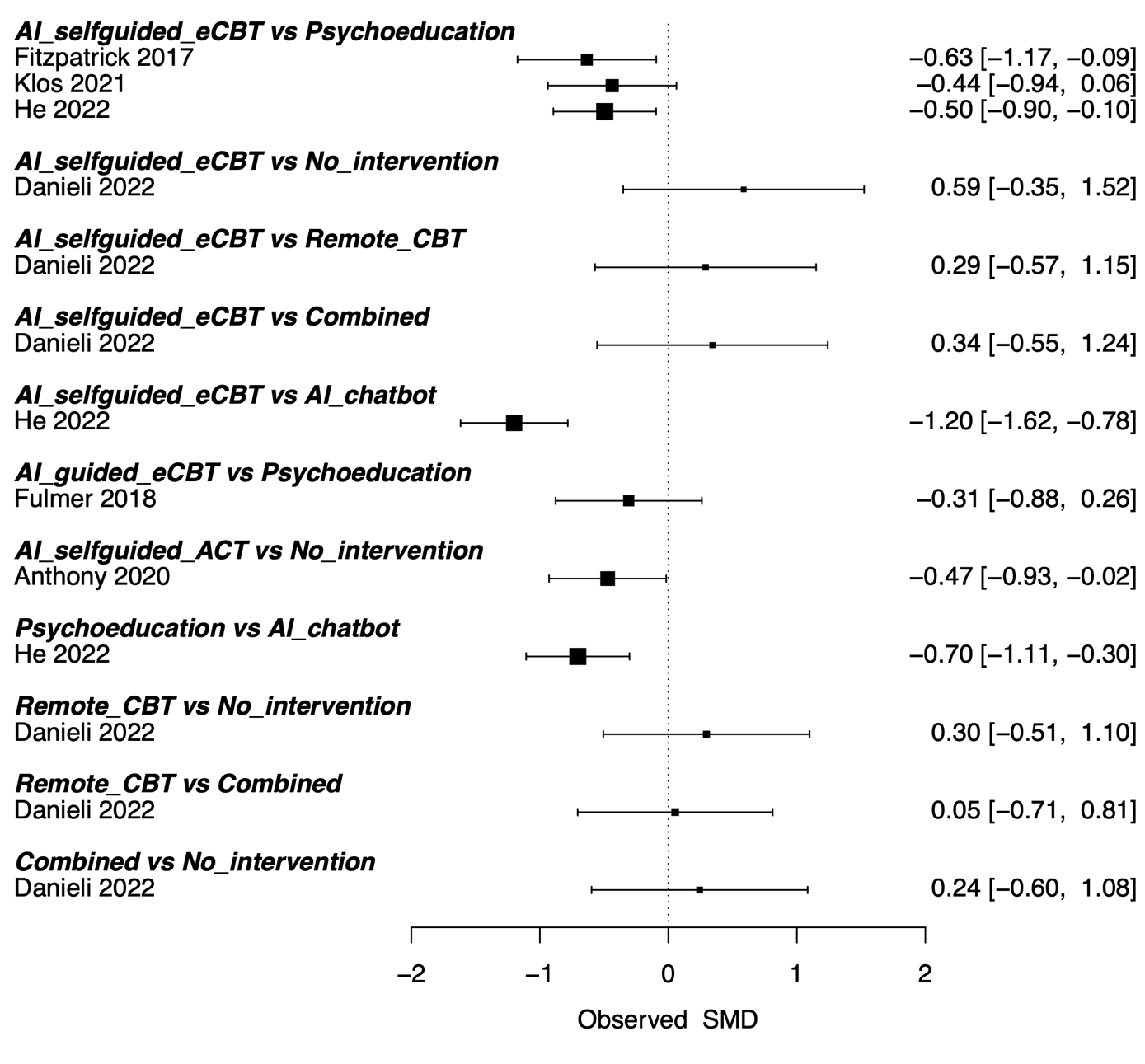


**Supplementary data 4:** Forest plots using a random effects model considering the depression scores in RCTs specific to the study of interventions for depression symptoms (n=4)


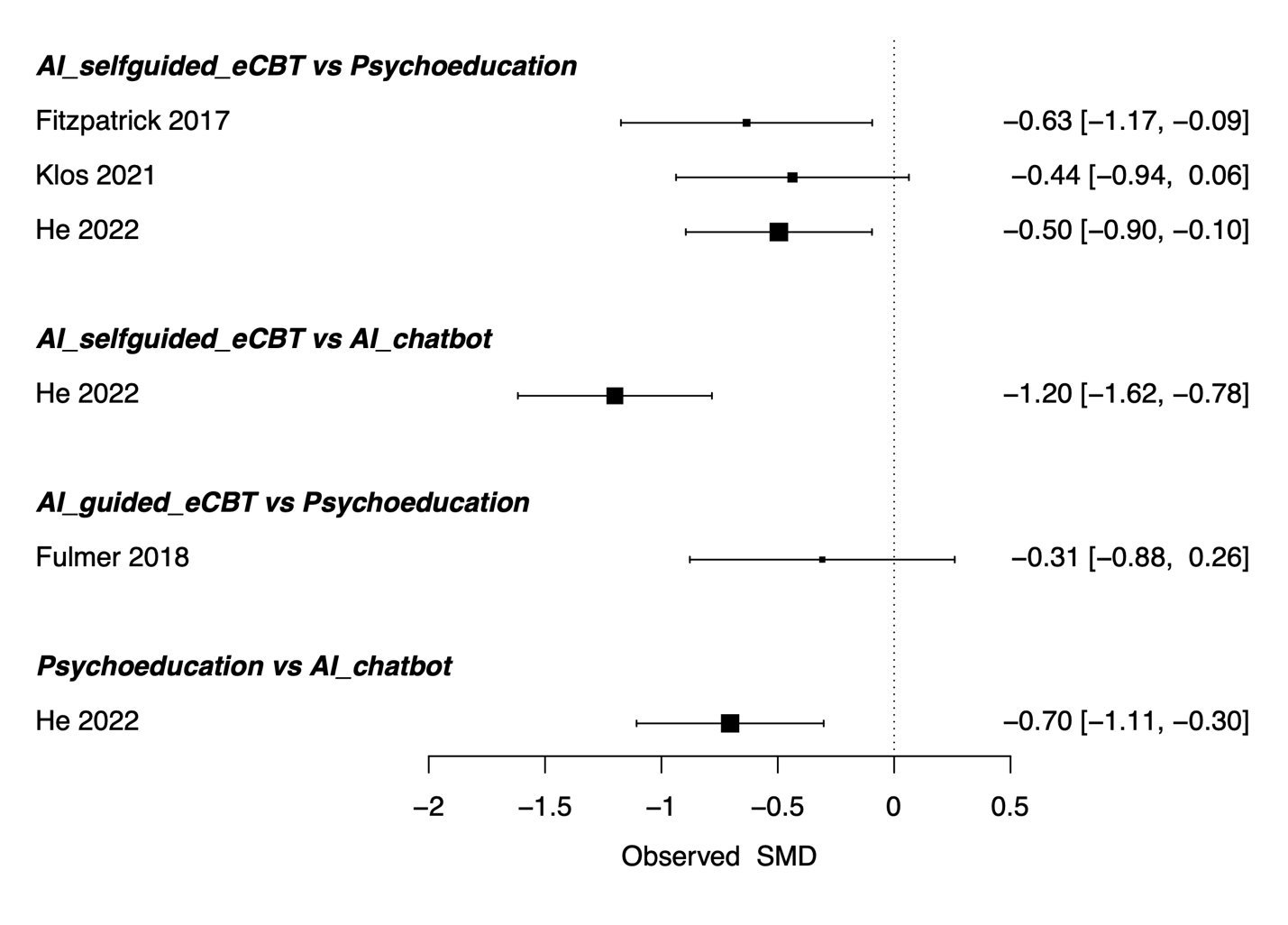


**Supplementary data 5:** Forest plots using a random effects model considering the anxiety scores in RCTs specific to the study of interventions for anxiety symptoms (n=4).


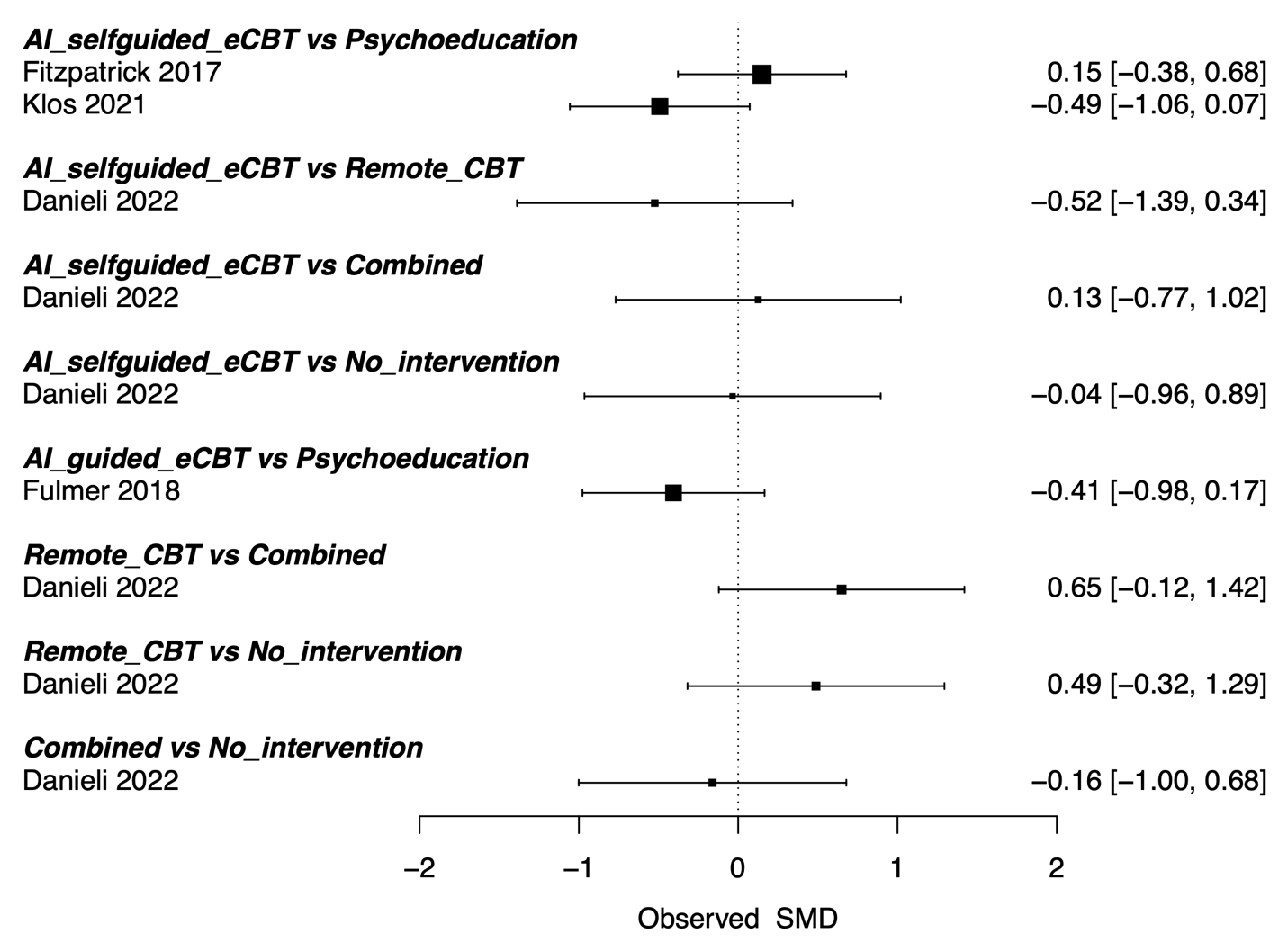


**Supplementary data 6:** Forest plots using a random effects model showing all RCTs reporting participant dropouts (n=6)


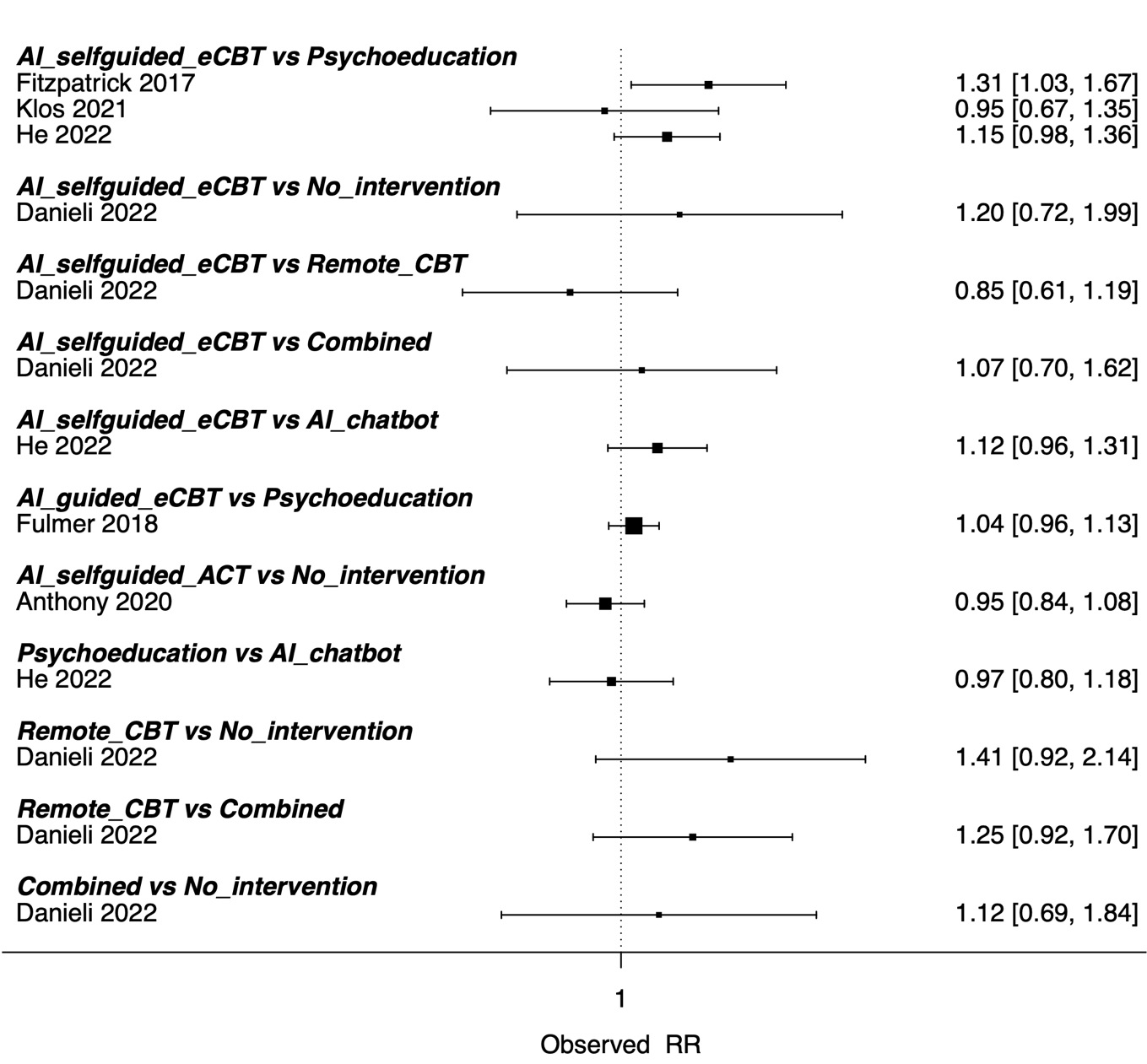


**Supplementary data 7:** Head-to-head network comparisons and ranking of intervention efficacy across all the RCTs included in this review. Interventions listed in the darker color diagonal with white letters: “AI self-guided eCBT”: AI agent delivering eCBT with no therapist guidance; “AI guided eCBT”: AI agent delivering eCBT with asynchronous therapist guidance; “Remote CBT”: Standard CBT delivered over the phone; “AI self-guided ACT”: AI agent delivered ACT with no therapist guidance; “Combined”: Remote CBT and AI self-guided eCBT; “AI chatbot”: AI agent providing general conversational responses not associated with CBT or therapy; “Psychoeducation”; and “No intervention”. Direct comparisons are present on the right side of the diagonal and indirect comparisons, on the left side of the diagonal. A-C presents SMD results, D presents RR results. A: Main effects of all the included RCTs (n=6), B: Depression scores in RCTs specific to the study of interventions for depression symptoms (n=4), C: Anxiety scores in RCTs specific to the study of interventions for anxiety symptoms (n=4), D: RCTs reporting participant dropouts (n=6)


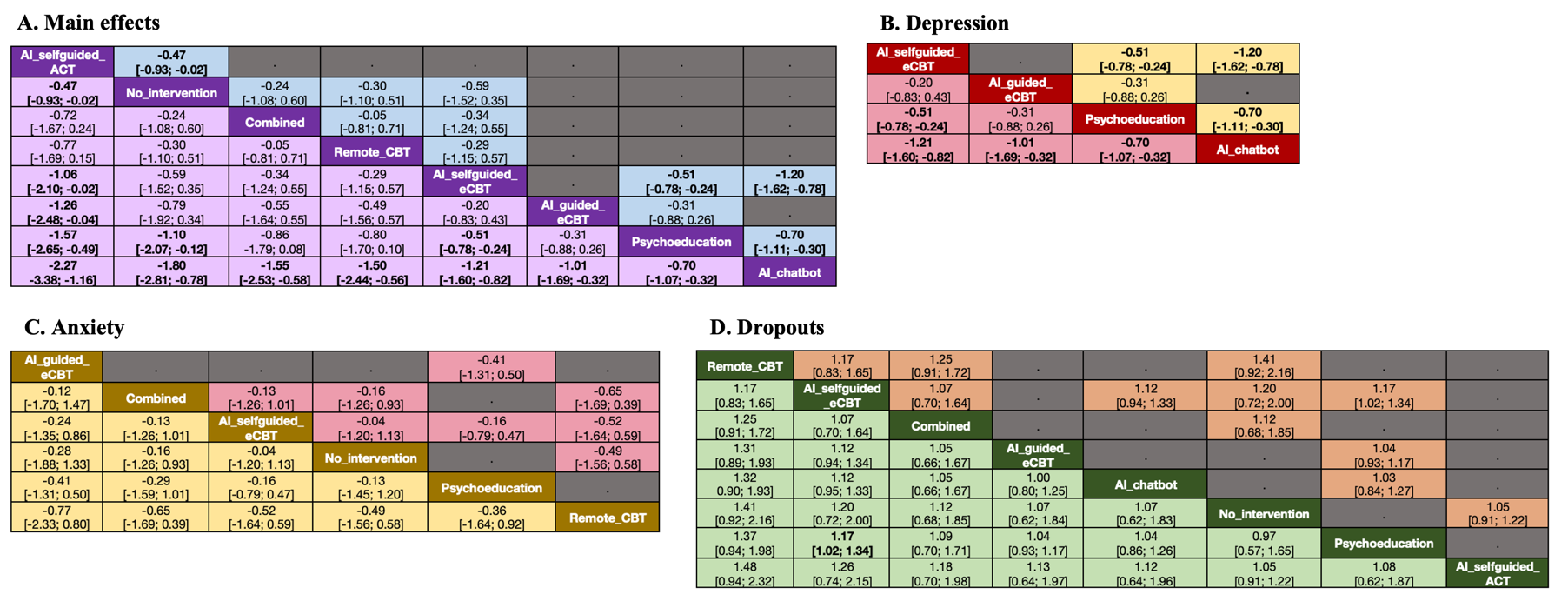


**Supplementary data 8:** Funnel plots – A: Main effects of all the included studies (n=10), B: Depression scores in studies specific to the study of interventions for depression symptoms (n=7), C: Anxiety scores in studies specific to the study of interventions for anxiety symptoms (n=4), D: Studies reporting participant dropouts (n=6)


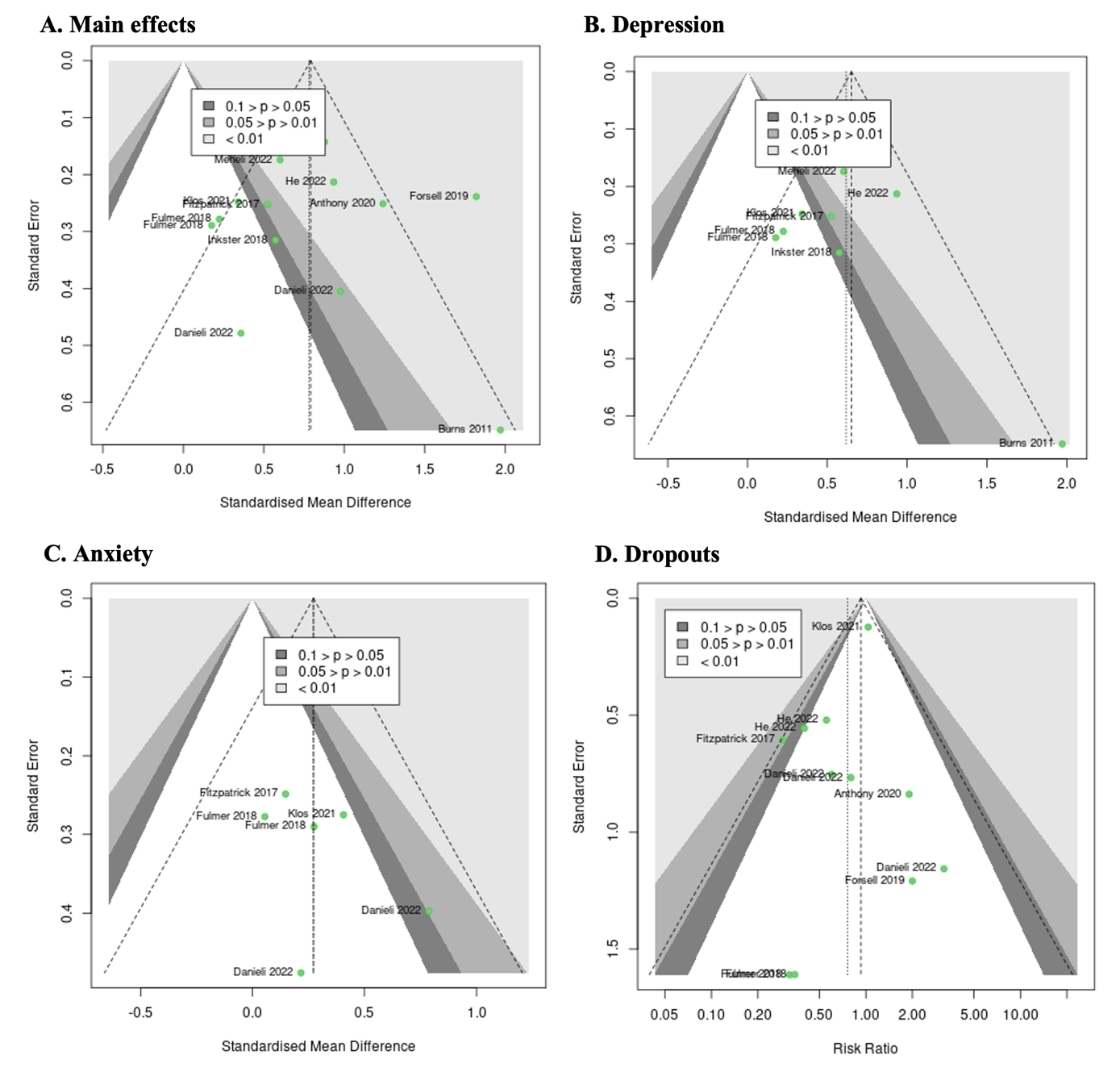

Supplement: Supplementary file 1 [file DataSheet_1.docx]
